# Echocardiography in Cardiac Assist Devices

**DOI:** 10.31083/j.rcm2307253

**Published:** 2022-07-14

**Authors:** Senthil Anand, Timothy Barry, Reza Arsanjani, Lisa LeMond

**Affiliations:** ^1^Department of Cardiology, Mayo Clinic Arizona, Scottsdale, AZ 85259, USA

**Keywords:** heart failure, echocardiography, cardiac assist devices, mechanical circulatory support

## Abstract

In patients with medically refractory heart failure or cardiogenic shock, both temporary and durable mechanical circulatory support devices can be used to support cardiac circulation. Both transthoracic echocardiography (TTE) and transesophageal echocardiography (TEE) are widely available, relatively noninvasive, and avoid radiation exposure. Thus, echocardiography is an invaluable tool that provides vital information aiding in preprocedure evaluation, placement, management, and weaning of cardiac assist devices. The purpose of this article is to review the utility of both TTE and TEE in managing patients with cardiac assist devices.

## 1. Introduction

Mechanical circulatory devices are increasingly used to help support patients with cardiogenic shock or end stage heart failure. This includes both temporary mechanical circulatory support (MCS) devices as well as durable circulatory support devices. Temporary MCS can be used as an escalation strategy, during high-risk interventional procedures, as a bridge to recovery, or as a bridge to durable MCS or transplant. Durable MCS can also be used as either a bridge to transplant or as destination therapy. Both transthoracic echocardiography (TTE) and transesophageal echocardiography (TEE) are widely available, reproducible, and relatively noninvasive. Hence TEE and TTE are frequently used to evaluate patients in whom mechanical circulatory support is being considered. Echocardiographic imaging can also aid in placement, management, and weaning of circulatory support devices. The purpose of this article is to review the role of both TTE and TEE in managing patients with cardiac assist devices.

## 2. Temporary Mechanical Circulatory Support Devices

At present, there are commercially available temporary MCS devices to support both the left ventricle as well as the right ventricle. Left-sided support devices include the intra-aortic balloon pump (IABP), Impella, and TandemHeart. Temporary right-sided devices include the Impella Right Percutaneous (RP) as well as the TandemHeart Protek Duo. The specifics of each of these devices will be discussed below.

## 3. Pre-Insertion Echocardiographic Evaluation of Temporary Left-Sided Circulatory Support Devices

Echocardiographic evaluation prior to insertion of temporary Left-sided devices isuseful to determine if temporary mechanical support would be beneficial to the patient, as well as to evaluate if there are any contraindications to use of any devices. Commonly used parameters of LV function include the ejection fraction (LVEF), stroke volume, as well as global longitudinal strain [1]. There are additional considerations specific to temporary left-sided support devices. These include the presence and severity of aortic regurgitation or stenosis, mechanical aortic valve, intracardiac thrombi, aortic dissection or plaques and intracardiac shunts. The presence of significant aortic regurgitation (AR) is a contraindication to use of intra-aortic balloon pumps (IABP) as they inflate during diastole which will worsen pre-existing AR [2]. While Impella devices can be used in the setting of aortic regurgitation, the regurgitation can worsen after placement of the device in a significant proportion of patients [3]. Severe aortic stenosis and mechanical aortic valves are contraindications to placement of Impella. The presence of LV mural thrombus and left atrial thrombus are contraindications of use of Impella and tandem hearts, respectively, as they can precipitate systemic embolization. Aortic dissection has been traditionally considered a contraindication to use of IABP as there were concerns over extension of the dissection flap. However, cases of its use in the setting of type A dissection with concomitant cardiac failure have been reported, and no adverse events were noted so long as TEE was utilized to confirm the wire and IABP placement to be in the true lumen [4]. Any pre-existing areas of possible shunting such as atrial septal defects (ASDs), patent foramen ovale (PFOs), and post myocardial infarction ventricular septal defects (VSDs) should also be noted as left-sided Impellas can precipitate right to left shunting resulting in refractory hypoxia [5].

## 4. Intra-Aortic Balloon Pump

**Fig. 1.**
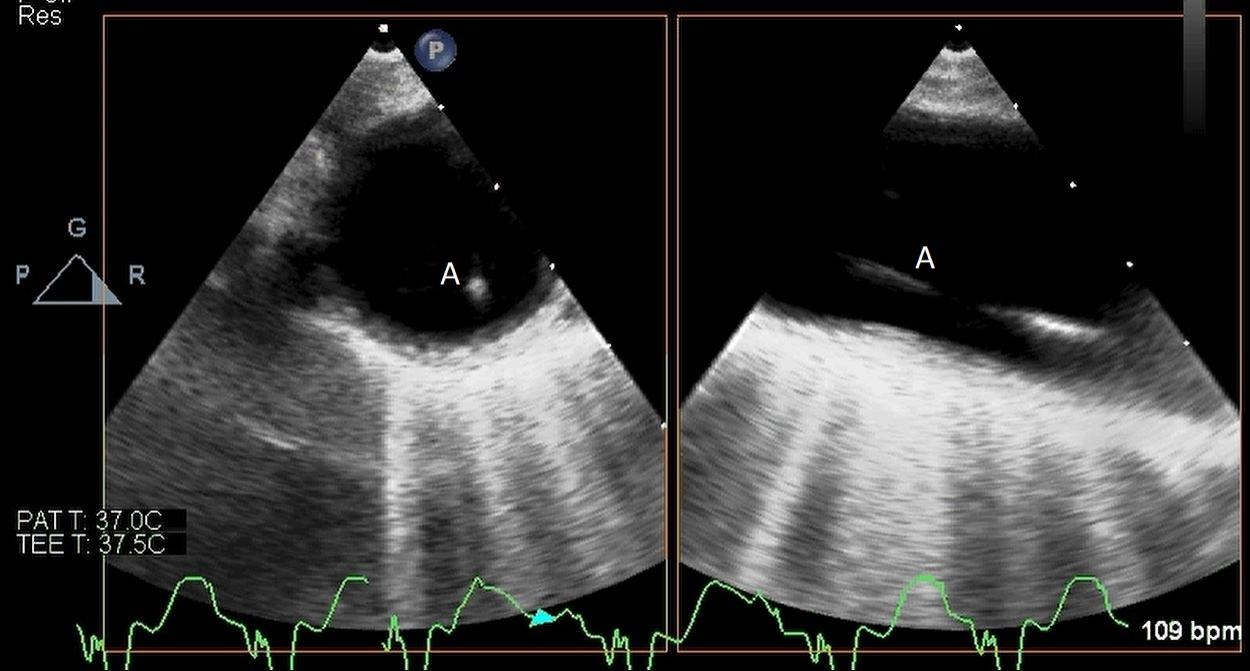
**TEE demonstrating IABP in descending aorta (A)**.

**Fig. 2.**
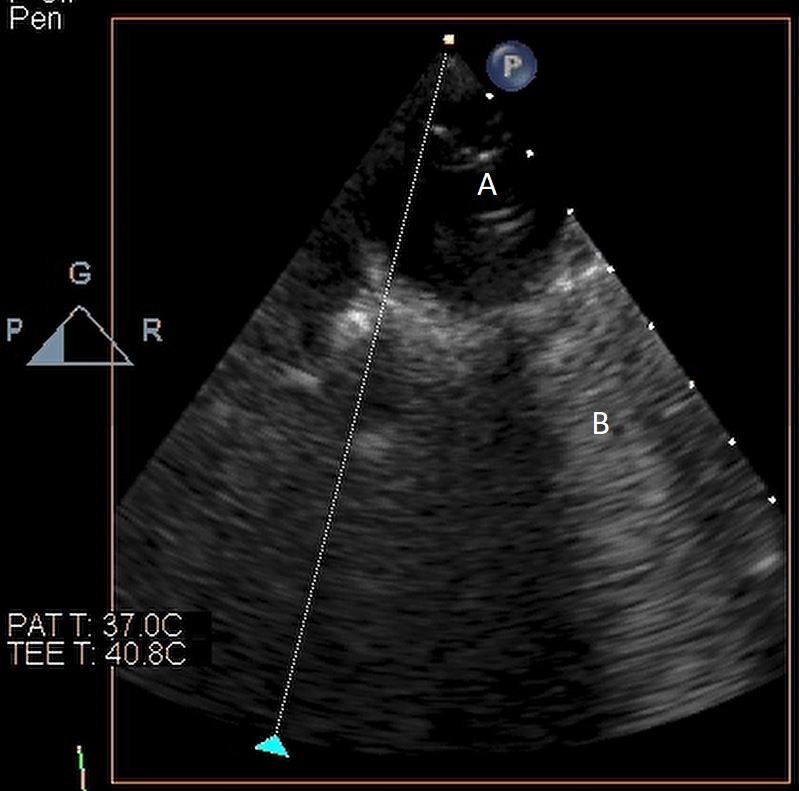
**TEE demonstrating IABP in descending aorta (A) with reverberation artifact seen behind it upon activation (B)**.

The intra-aortic balloon pump is a percutaneously placed counter pulsation device which helps in decreasing afterload as well as augmenting coronary perfusion. Initially developed in the 1960s it is the oldest MCS device and given its simplicity, cost effectiveness, and ease to implant and explant, it is the most commonly used temporary support device [6]. Although it is typically placed in the cardiac catheterization lab under fluoroscopic guidance, TEE can be utilized to help in its placement in the intubated patient in the intra-operative setting. The femoral artery is the most common site of placement however they can on occasion be placed in alternative sites such as the axillary artery or directly into the aorta [7, 8]. When placed via the femoral artery, it is threaded over a guidewire. TEE can be used to visualize both the guidewire as well as the tip of the IABP catheter during placement (Fig. 1) [9]. Ideal positioning of the balloon tip is 1–2 cm distal to the left subclavian artery to derive maximal hemodynamic benefit [10]. Positioning can be confirmed by visualizing the descending aorta and then withdrawing the TEE probe until the left subclavian artery and aortic arch are visualized. Upon activation of the balloon pump the gas filled balloon will cause shadowing and reverberation artifacts (Fig. 2). Its presence can be used as confirmation of proper function of the device. If these artifacts are not seen or bubbles are visualized in the aorta, rupture of the IABP should be suspected [9]. In addition to hemodynamic monitoring with a Swan-Ganz catheter, TTE can be used to monitor LV function after IABP placement and can help guide weaning of IABP support. It can also visualize any new or worsening aortic regurgitation. Given that IABPs work by reducing afterload, on rare occasions they can precipitate dynamic outflow tract obstruction and paradoxically worsen cardiogenic shock. Examples include patients with a relatively preserved basal or septal myocardial function in scenarios such as takotsubo cardiomyopathy or acute myocardial infarctions. Doppler imaging and color flow doppler can be used to identify such scenarios [11].

## 5. Impella

The Impella is a catheter-based device which is placed in the LV across the aortic valve. It uses a mechanical Archimedes screw to pump blood from the LV into the aorta, thereby immediately unloading the LV and increasing cardiacoutput [12]. Current commercially available left-sided Impellas include the Impella 2.5, CP (Cardiac Power), 5.0, 5.5, and LD (Left Direct). Typically smaller devices such as the 2.5 or CP are placed percutaneously through the femoral artery while larger devices with higher flow rates such as the 5.0 or 5.5 are placed via either a surgical graft into the axillary artery or less commonly the femoral artery via cut down. Like IABP, Impella devices are commonly placed under fluoroscopic guidance, but when available TEE can offer additional information to aid in device positioning and function [13]. Cases in which bedside placement of Impellas done with TEE guidance alone have been reported. This strategy can be considered in patients with refractory shock preventing transportation of the patient to the cardiac catheterization lab. In one single center, retrospective study describing cases in which TEE alone guided placement was utilized for 55 patients there was no difference in Impella-related complications when compared with the fluoroscopic guided cohort of 95 patients [14].

**Fig. 3.**
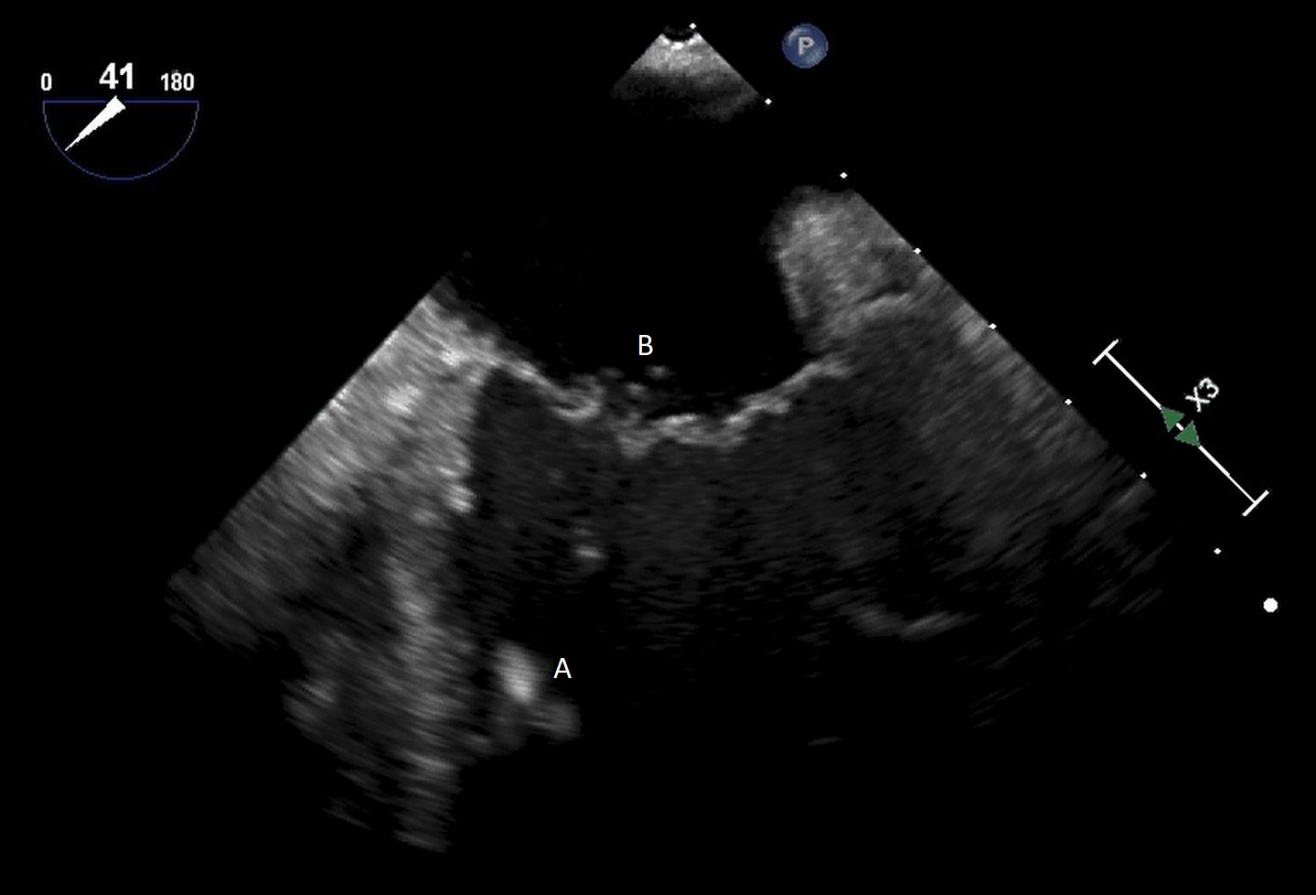
**Impella (A) in the LV cavity caused disruption and damage to subvalvular apparatus resulting in flail segment (B) of the mitral valve**.

**Fig. 4.**
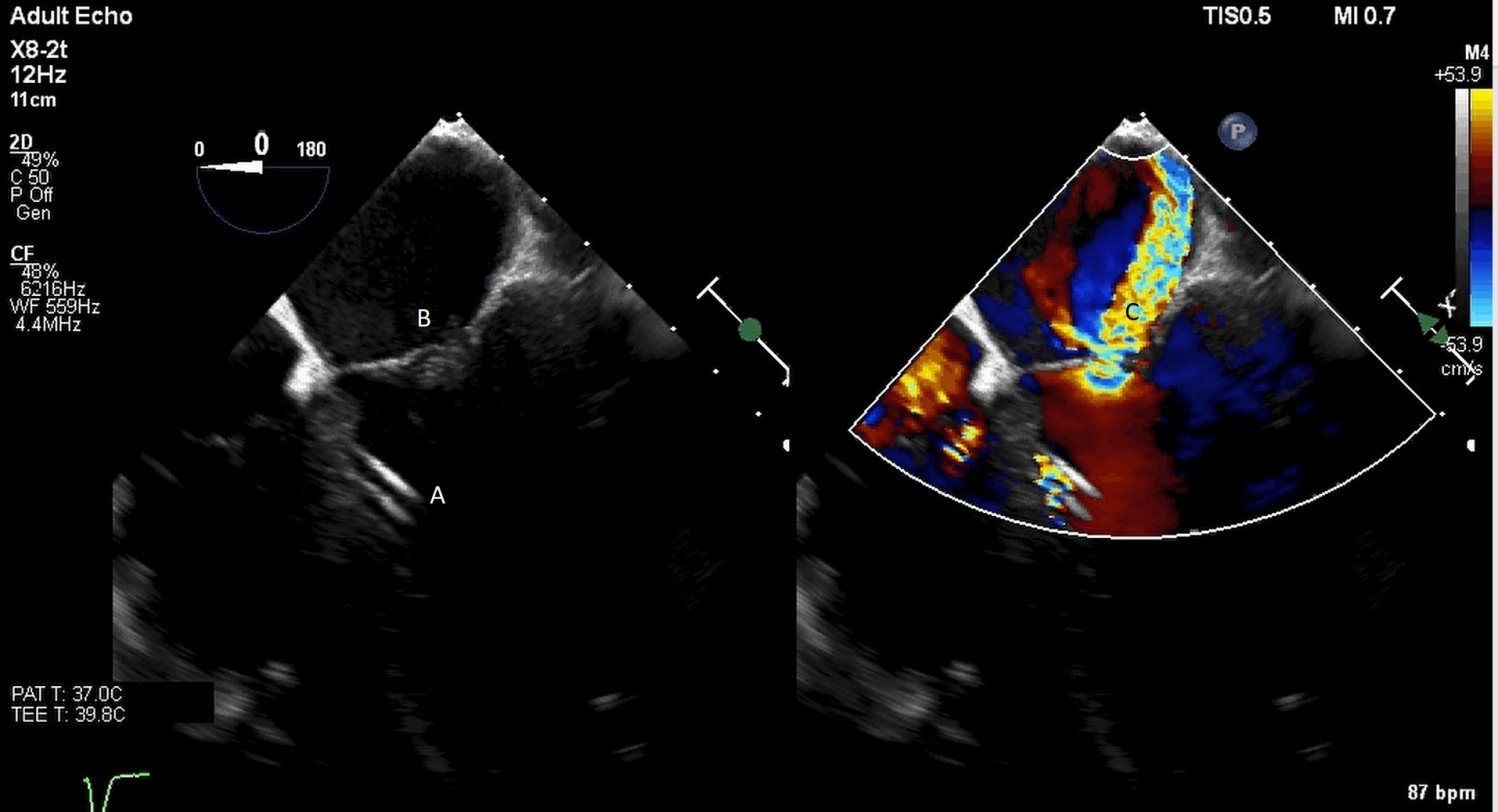
**Impella (A) placement causing disruption and damage to subvalvular apparatus resulting in mitral valve flail (B) and mitral regurgitation (C)**.

After initial access is obtained with a guidewire, TEE can confirm placement of the guidewire within the aorta and ensure there is no iatrogenic dissection from the procedure. The midesophageal long axis and 4 chamber views can be used to visualize the guidewire crossing of the aortic valve and positioning within the LV cavity. The wire tip should point towards the LV apex. Wire placement too deep within the LV can trigger ventricular arrhythmias and tethering of the mitral valve or subvalvular apparatus should be avoided as this can result in the inlet abutting the mitral valve or damage to subvalvular apparatus (Figs. 3,4) [13]. When the proceduralist is advancing the Impella over the guidewire, the best view to observe the device crossing the aortic valve is the midesophageal long axis view [13].

**Fig. 5.**
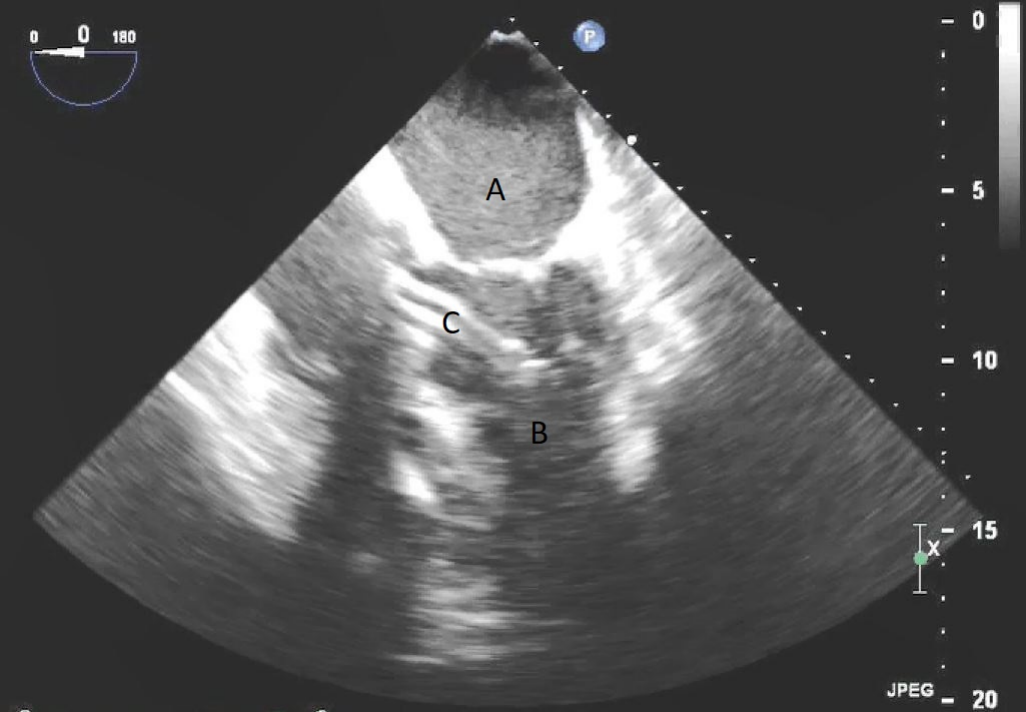
**A midesophageal 4 chamber view on TEE demonstrating an Impella traversing the aortic valve with the inflow port in the left ventricle**. (A) Left Atrium. (B) Left Ventricle. (C) Impella.

**Fig. 6.**
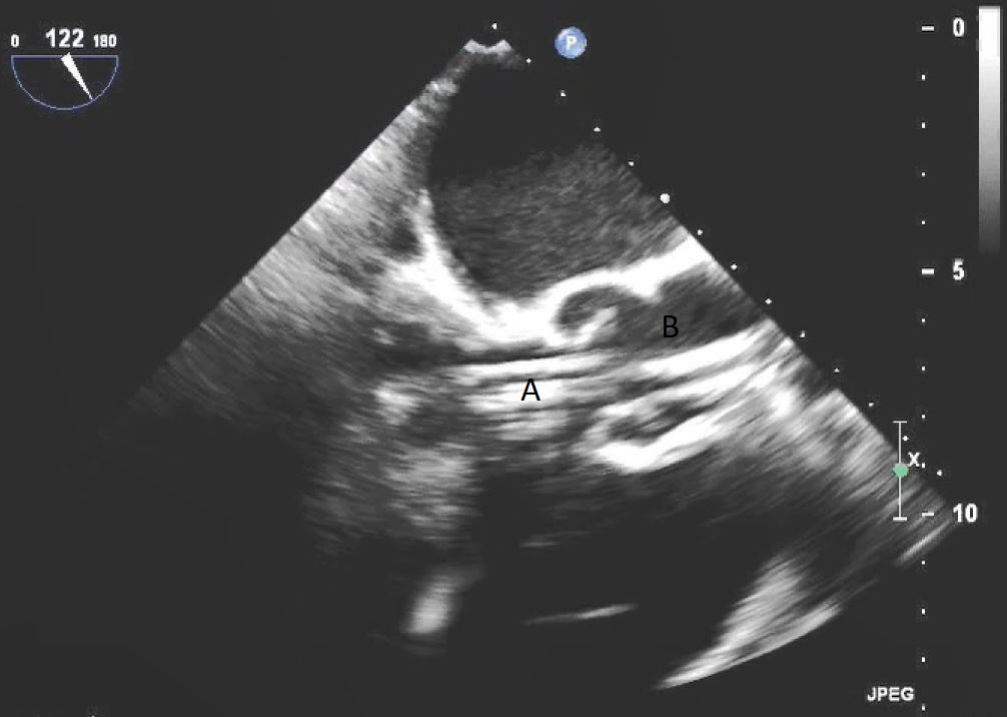
**A midesophageal long axis view zoomed up on the aortic valve demonstrating the Impella traversing an open aortic valve**. (A) Impella. (B) Ascending aortic root.

**Fig. 7.**
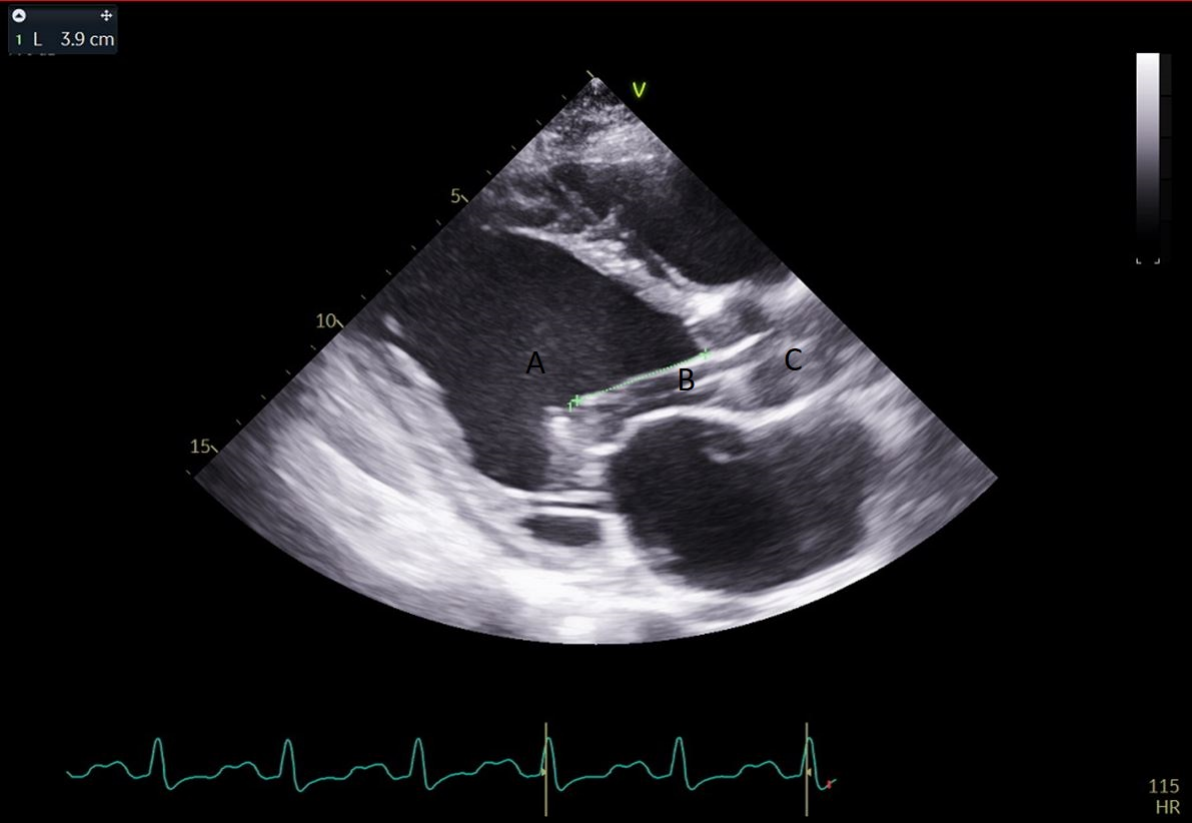
**A parasternal long axis view on a transthoracic echocardiogram**. The distance from the Impella inlet to the aortic valve is measured and noted to be 3.9 cm. (A) LV Cavity. (B) Impella. (C) Ascending aortic root.

**Fig. 8.**
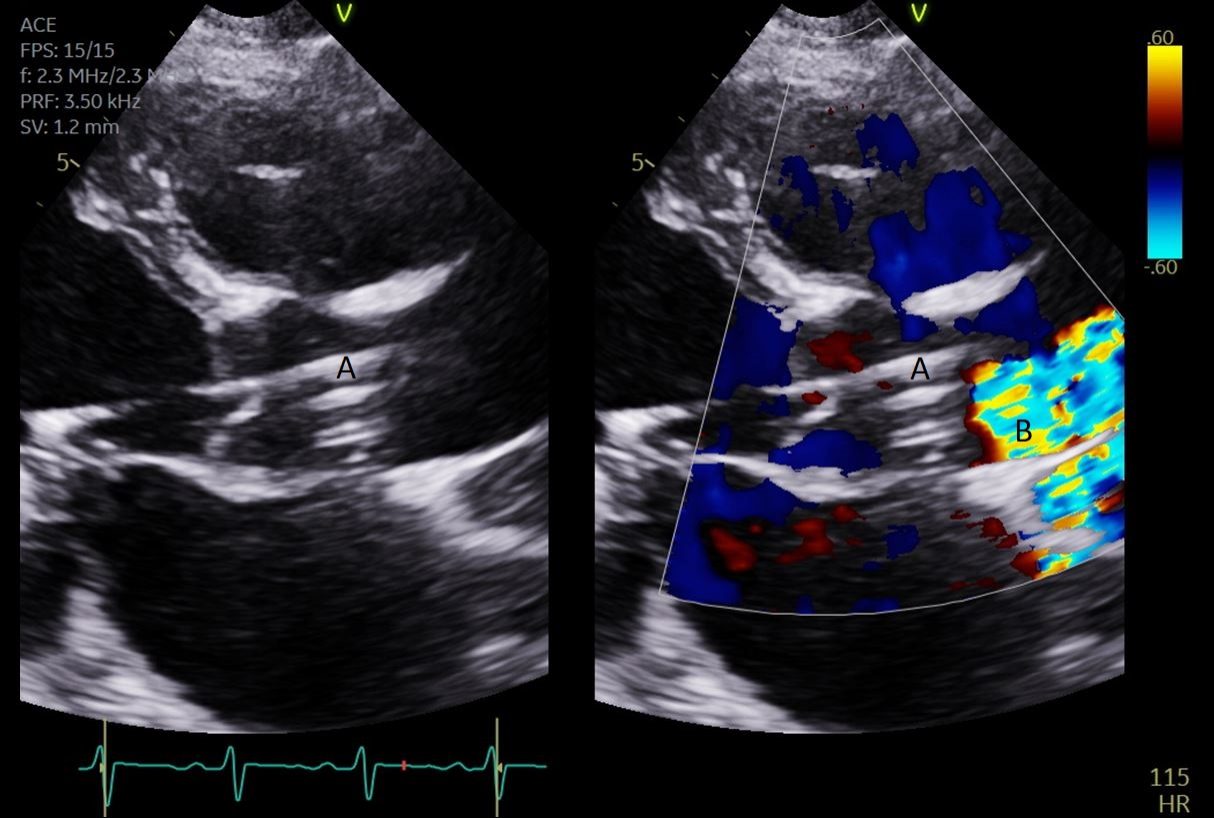
**A parasternal long axis view on a transthoracic echocardiogram zoomed up on the aortic valve**. Color flow imaging demonstrates a mosaic pattern at the Impella outlet, confirming its position as being above the aortic valve. (A) Impella outlet. (B) Mosaic pattern at Impella outlet on color flow doppler.

**Fig. 9.**
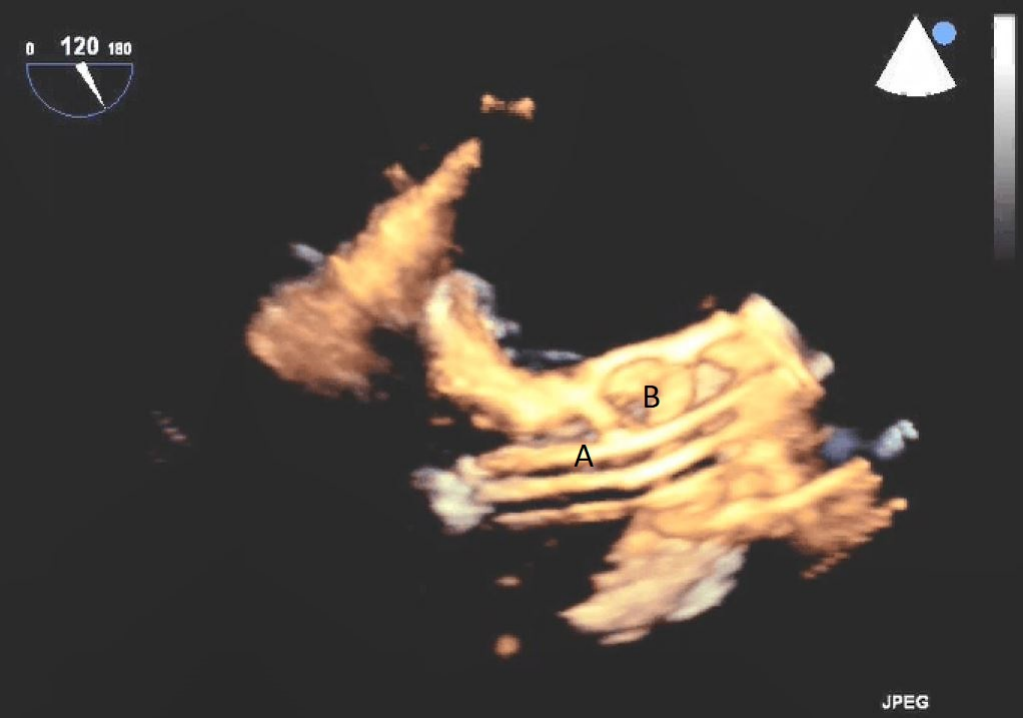
**Real time 3D TEE imaging visualizing the Impella in relation to the aortic valve and LVOT**. (A) Impella. (B) Ascending aortic root.

Both TTE and TEE can help with ideal positioning of the Impella (Figs. 5,6). The distance from the aortic valve to the Impella inlet should be measured. This should ideally be 3.5–4 cm for all Impella devices except for the Impella 5.5 for which it is 5 cm [15] (Fig. 7). The outlet should be 1.5–2 cm above the sinuses of Valsalva. The catheter should be angled towards the LV apex and away from the septum and mitral valve. The positioning of both the inlet in the LV cavity and the outlet above the aortic valve should be confirmed. Color flow doppler imaging can help confirm this positioning as a mosaic pattern will be visualized near the inlet and outlet ports on spectral doppler (Fig. 8). Real-time 3D echocardiography can also be used to help in visualizing Impella positioning relative to other anatomical structures (Fig. 9). After placement of the Impella, the aortic and mitral valves should be interrogated for any new or worsening regurgitation or dysfunction [16]. TEE can also help identify additional complications of Impella placement including pericardial effusion or LV free wall rupture [17].

**Fig. 10.**
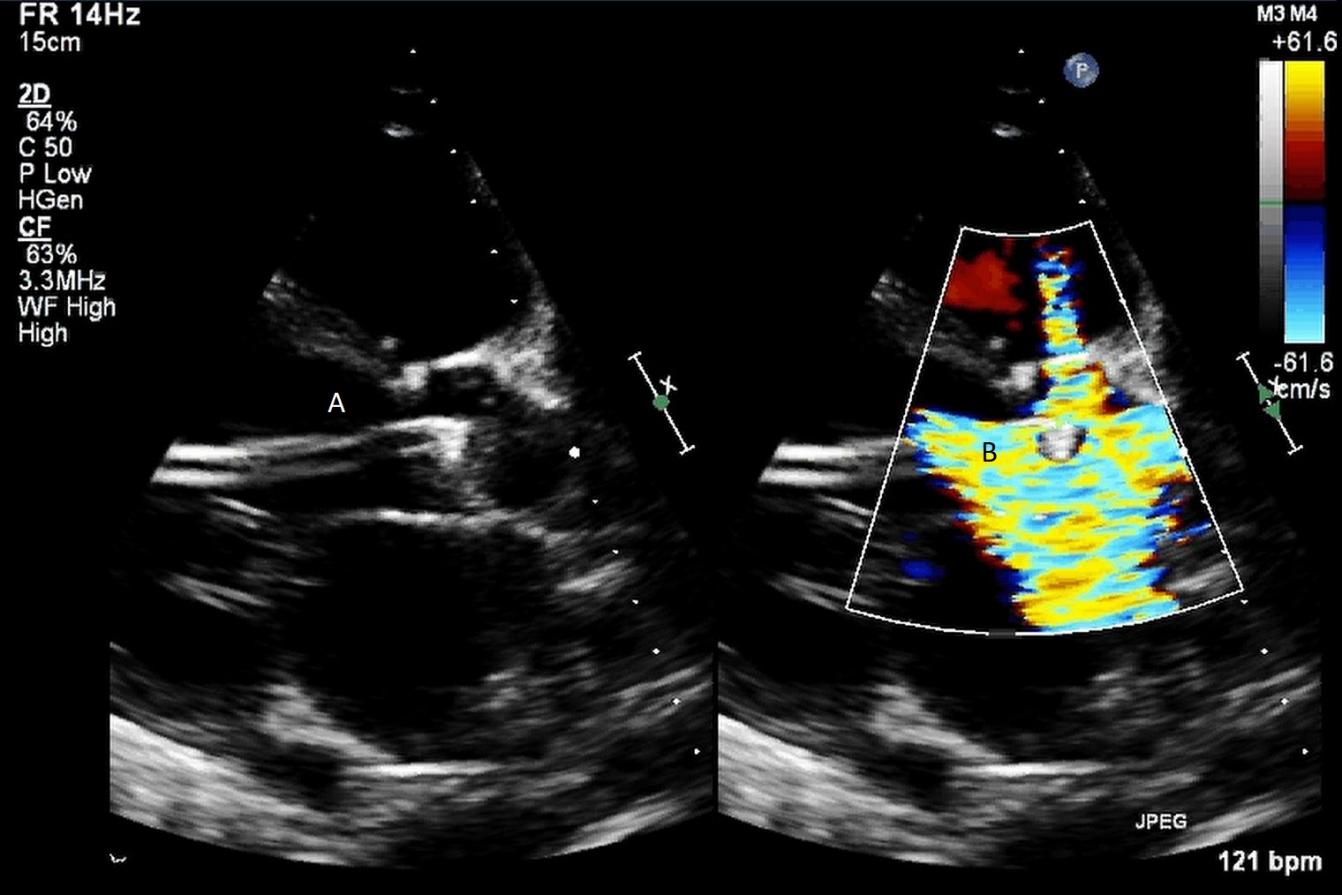
**Impella (A) visualized on parasternal long axis view on TTE**. Color flow imaging shows that the impella outlet is too low (B) as the mosaic pattern at the outlet is seen in the LVOT beneath the aortic valve.

TTE can be used to monitor the Impella and LV function periodically after successful placement. The ideal septal position is midline during diastole and shifts in septal position can indicate the need to manage concomitant RV failure or adjust Impella speeds as needed. The Impella device can also migrate, resulting in both the inlet and outlet ports being positioned on the same side of the aortic valve (Fig. 10). This will cause recirculation and failure to provide adequate circulatory support [18]. Finally, echocardiographic data can be used in conjunction with invasive hemodynamic data to help with weaning of the Impella by evaluating the response of the LV to progressive reduction in the support provided by the Impella (the P level). Assessment of intrinsic heart function is feasible at minimal flow support (P2) [19]. Dobutamine stress echocardiography can also be utilized to evaluate contractile reserve and help predict successful weaning of Impella [19]. In patients with poor residual contractility or no contractile reserve, evaluation for durable mechanical circulatory support devices or transplantation candidacy should be considered.

## 6. TandemHeart

The TandemHeart is an external centrifugal pump attached to a percutaneously placed cannula utilized to pump blood from the left atrium into the aorta [20]. A 21 French venous cannula is placed in the femoral vein, and drains oxygenated blood from the left atrium via a transseptal puncture. This blood is delivered to the aorta via a 17 French cannula placed in the femoral artery [21]. Similar to other temporary mechanical circulatory support devices, placement is often under fluoroscopic guidance, but when available, TEE offers additional information to aid placement.

When using TEE, a midesophageal bicaval view can be utilized to assist transseptal puncture [22, 23]. Initially the guidewire is advanced from the femoral vein into the right atrium (RA). The wire is then replaced by a transseptal puncture needle within a catheter tip. This should be angled towards the thinnest part of the interatrial septum (IAS), the fossa ovalis. This can be identified by rotating between the bicaval and the midesophageal aortic valve short axis views. When the transseptal needle is advanced in this location, tenting of the IAS into the left atrium can be visualized and once positioning is confirmed, transseptal puncture can be completed [23]. The catheter is advanced into the left atrium and the needle is exchanged for a guidewire which is positioned in the left upper pulmonary vein. This can be visualized in the mid-esophageal 4 chamber view with the transducer rotated to visualize both left pulmonary veins. Finally, a sheath and dilator are advanced into the left atrium over the guidewire and the inflow cannula is positioned in the left atrium. Utilizing TEE helps lower the risk of complications such as puncturing the aorta or left atrial wall [24]. Additional complications such as tamponade can also be immediately identified if TEE is utilized during the procedure [25]. As noted previously, the arterial cannula is placed in the femoral artery. TEE can be used to confirm guidewire position but cannot visualize the inflow cannula positioning as this is in the iliofemoral artery. Upon activation of the TandemHeart, color flow doppler can help visualize the inflow cannula in the left atrium , as well as confirm that there is no blood being drawn from the right atrium [26].

TTE can be used to monitor LV systolic function while being supported by the TandemHeart. Since the inflow cannula is in the left atrium, and the outflow is in the aorta, the LV will be underfilled. It is important therefore to also monitor for residual LV function and ensure that the aortic valve is opening. Lack of aortic valve opening increases risk of LV or aortic root thrombus [26].

## 7. Echocardiographic Evaluation Prior to Insertion of Temporary Right-Sided Circulatory Support Devices

A combination of hemodynamic data from Swan-Ganz catheterization and echocardiography parameters can be used to identify patients that are in right ventricular (RV) failure and those who would potentially benefit from right-sided support devices. Quantitative measures suggested by the American Society of Echocardiography (ASE) to evaluate RV function include tricuspid annular plane systolic excursion (TAPSE, normal ≥18 mm), RV tissue Doppler S velocity (normal >10 cm/s), and RV fractional area change (RVFAC, normal >35%) [27]. Additional measures that can be used include RV free wall strain (normal is more negative than –25%) and 3D evaluation of RV systolic function [28].

When considering utilizing RV support devices for patients in RV failure, TTE and TEE can help identify any contraindications to device placement or issues which can result in device dysfunction. This includes thrombi in the RA or RV, tricuspid or pulmonary valve stenosis, significant pulmonary regurgitation, mechanical prosthesis, or intracardiac shunts (ASD, VSD, or PFO) which can result in left to right shunting.

## 8. Impella Right Percutaneous (Impella RP)

**Fig. 11.**
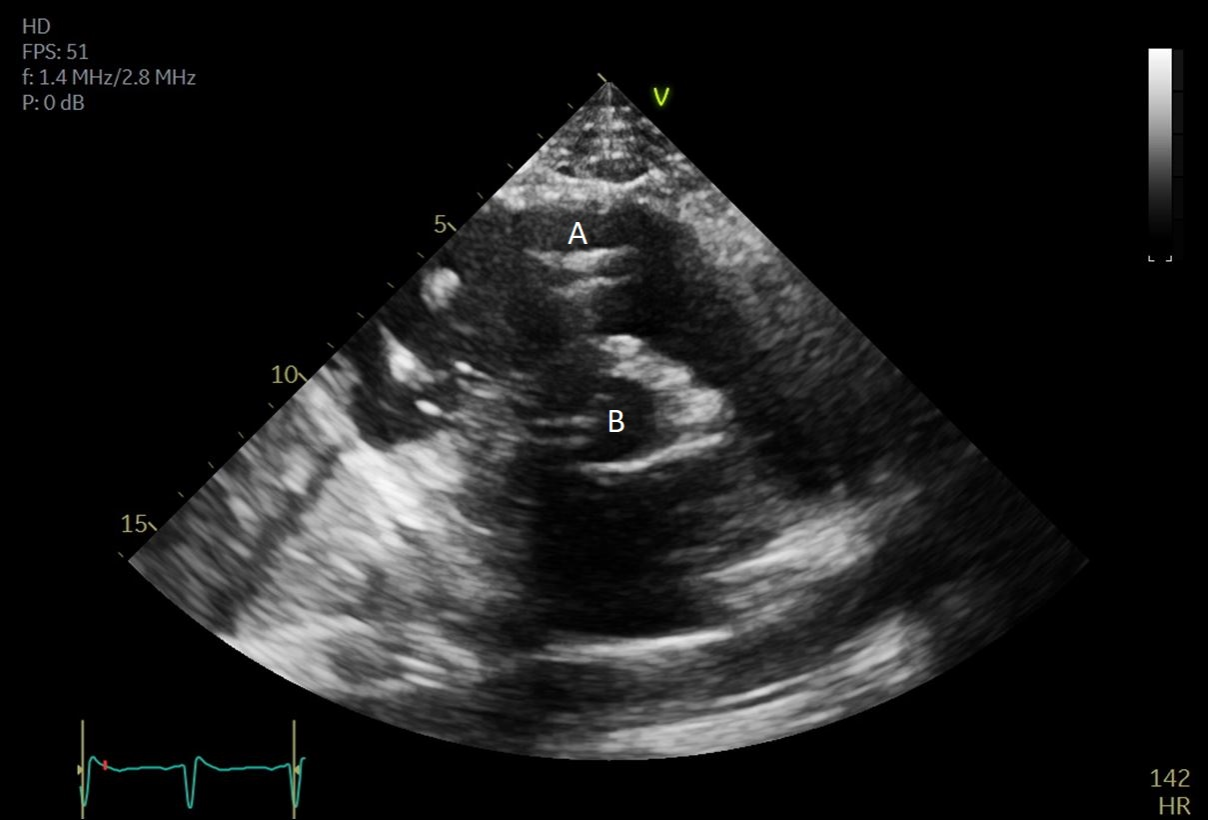
**A parasternal short axis view at the level of the aortic valve on a transthoracic echocardiogram**. The Impella RP devices is seen in the RVOT. (A) Impella RP in the RVOT. (B) Aortic valve with a left sided Impella seen traversing it.

**Fig. 12.**
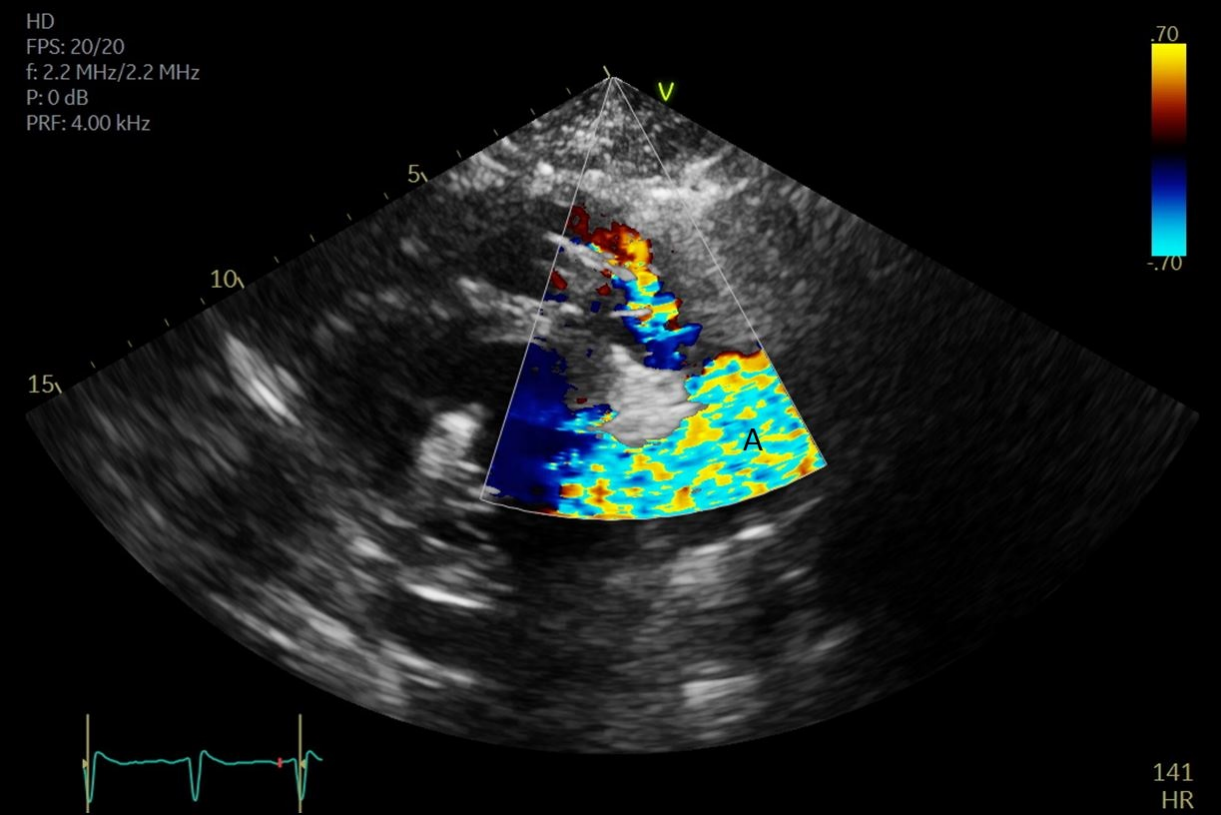
**Color flow imaging demonstrating a mosaic pattern at the Impella RP outlet, confirming its position in the main pulmonary artery**. (A) Mosaic pattern in the main pulmonary artery on color flow dopper.

The Impella RP is a percutaneous micro-axial propeller pump which is placed via a 23 French sheath into the femoral vein. It is positioned across the tricuspid and pulmonary valves with the inflow port in the inferior vena cava (IVC) and the outflow in the pulmonary artery (PA). Ideally, the outflow port is in the main pulmonary artery pointed towards the left PA so the Swan-Ganz catheter can be positioned in the right PA [29]. A bicaval view on TEE can be used to visualize the inflow port which is typically positioned at the IVC/RA junction. A midesophageal RV inflow-outflow view and upper esophageal views can be used to confirm appropriate outflow port positioning [29]. Similar to left-sided Impella devices, the Impella RP can also migrate and result in device malfunction. If the outflow port is positioned at the level of or below the pulmonary valve, this can result in reduced support and even recirculation completely within the RV. If this is suspected, TTE or TEE can be utilized to verify Impella positioning [30] (Figs. 11,12).

## 9. TandemHeart Protek Duo (TPD)

**Fig. 13.**
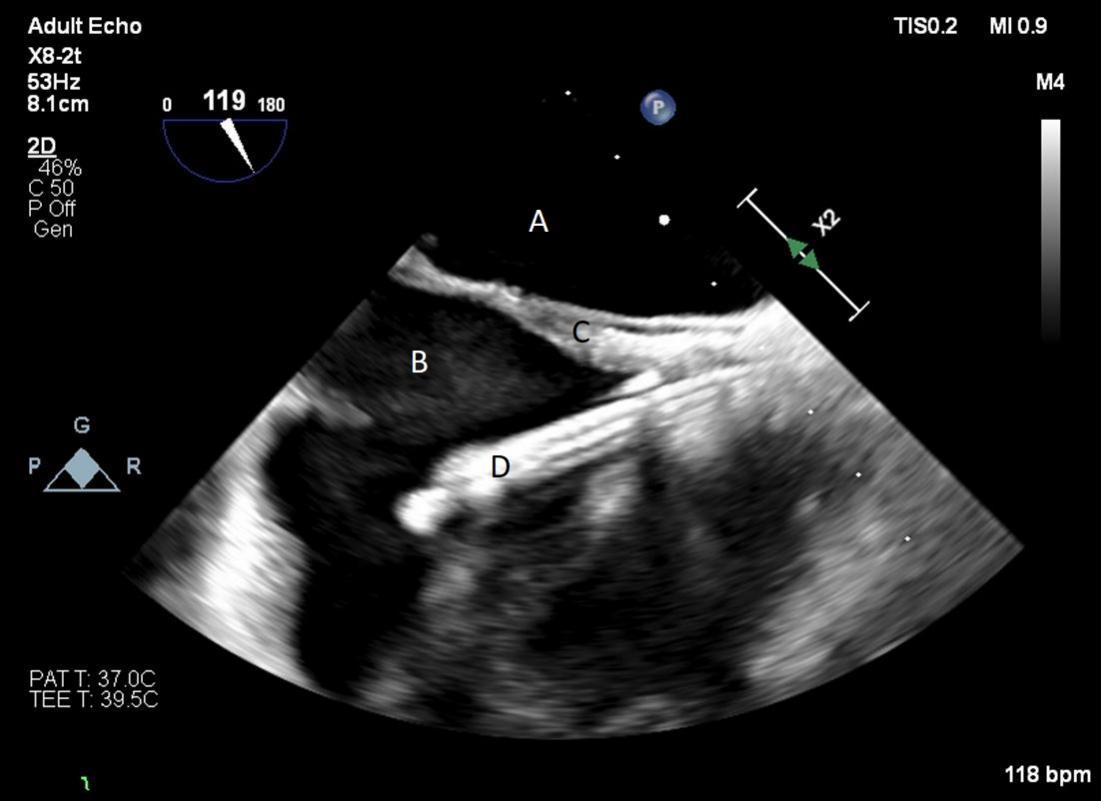
**A bicaval view on a TEE done during placement of a Protek Duo**. The inflow lumen is seen entering the right atrium from the SVC. (A) Left atrium. (B) Right atrium. (C) Interatrial septum. (D) Protek Duo inflow lumen.

**Fig. 14.**
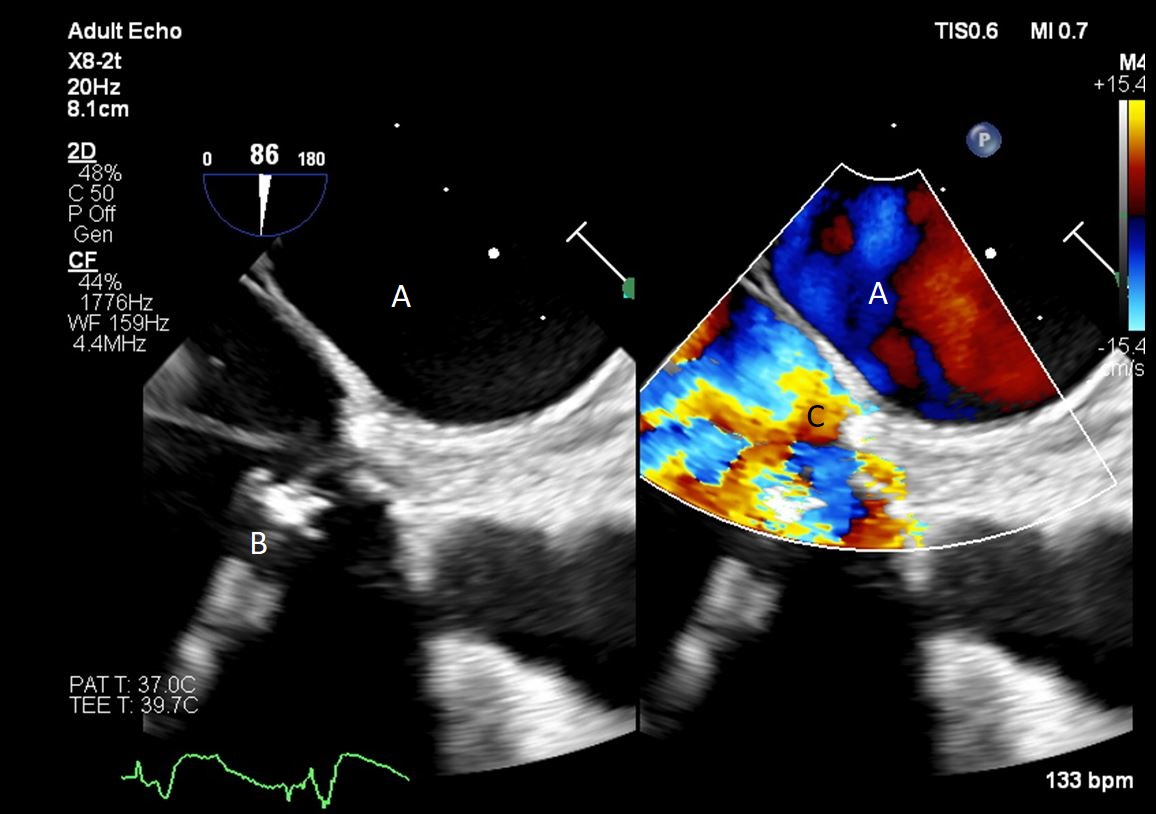
**Color flow imaging demonstrating a mosaic pattern at the inflow port**. The interatrial septum and left atrium are also visualized. Note that no blood flow is being entrained from the left atrium across the inter atrial septum. (A) Left atrium. (B) Inflow port in the right atrium. (C) Mosaic pattern at the inflow port on color flow doppler.

**Fig. 15.**
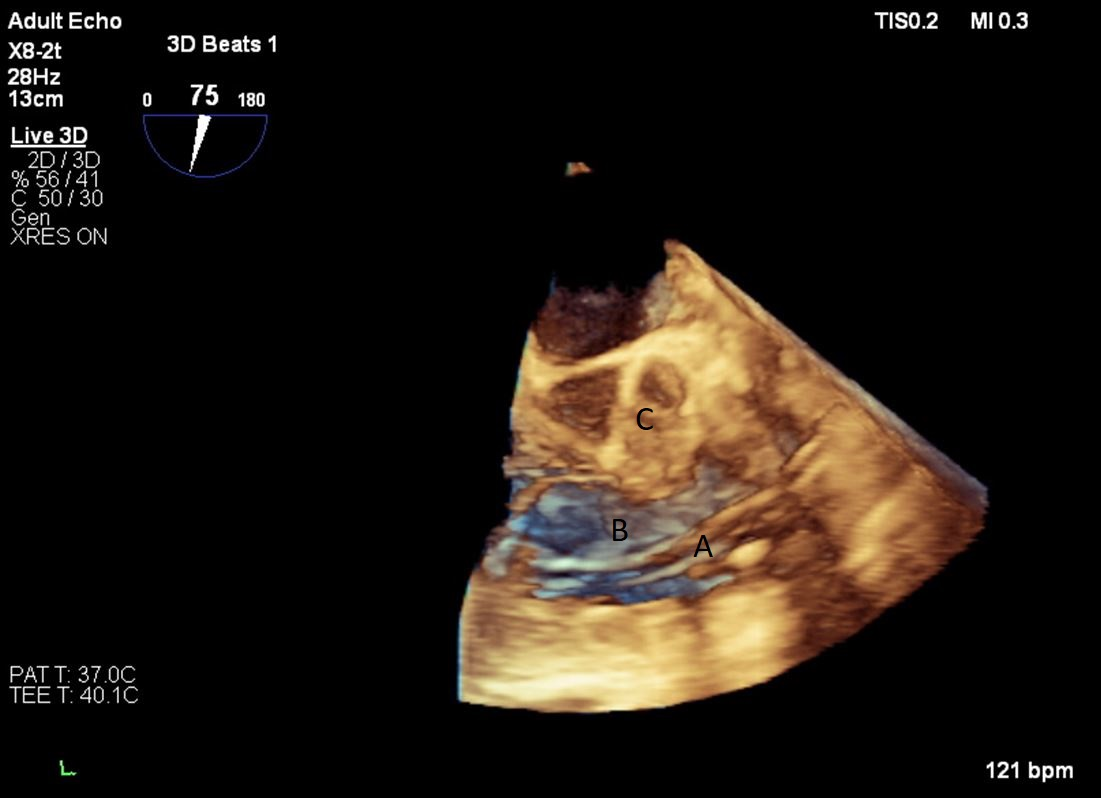
**Real time 3D TEE imaging of the RV inflow outflow view demonstrating the Protek Duo in the RVOT**. (A) Protek Duo. (B) RVOT. (C) Aortic valve.

**Fig. 16.**
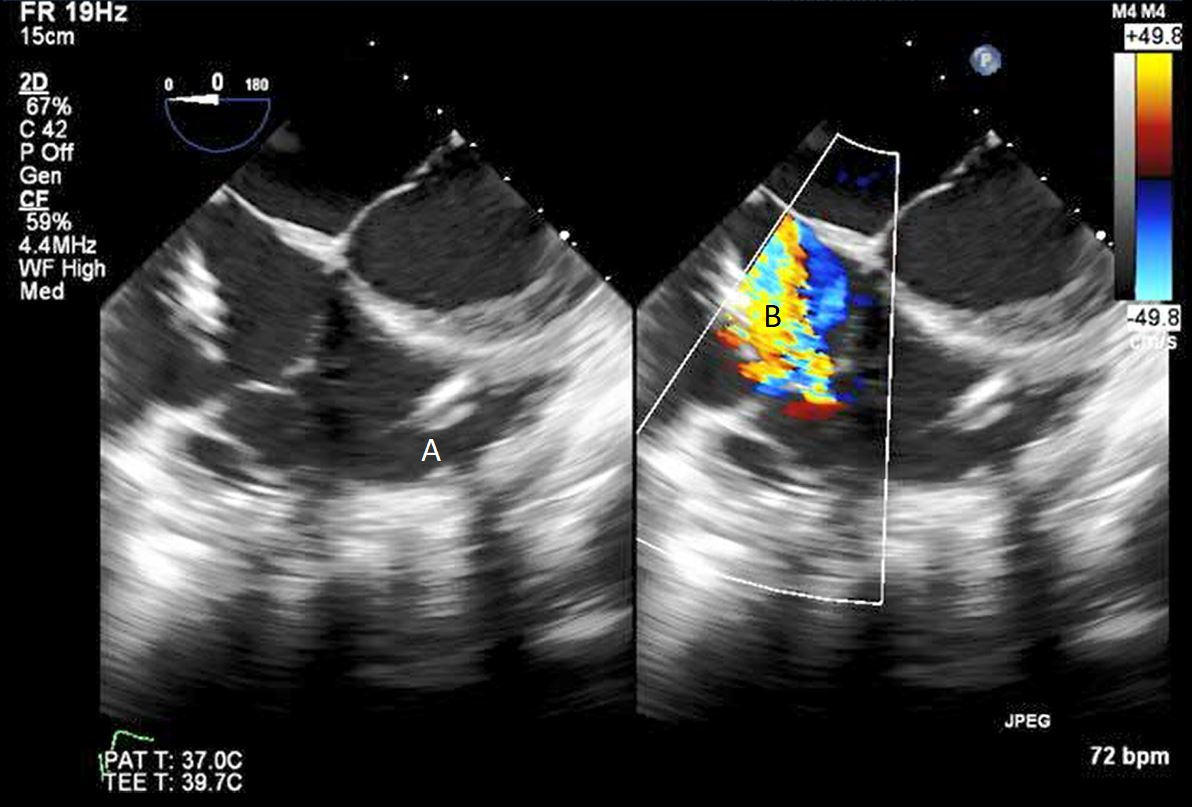
**Protek Duo (A) visualized in RV**. Its placement caused disruption of tricuspid valve with resultant tricuspid regurgitation seen on color flow imaging (B).

The TandemHeart Protek Duo is a percutaneous right ventricular assist device (RVAD) placed via a dual-lumen 29 French sheath in the right internal jugular vein. The inflow lumen is situated in the right atrium and outflow lumen in the main pulmonary artery. The port lumens are connected externally to a TandemHeart centrifugal pump [31]. As this is generally placed in the operating room, intra-operative TEE can be used to help guide placement. Similar to the Impella RP, bicaval and midesophageal 4 chamber views can visualize the inflow cannula and RV inflow-outflow view and upper esophageal views can be used to visualize the outflow cannula (Figs. 13,14,15). On occasion, its placement can result in distortion of the tricuspid valve morphology with resultant tricuspid regurgitation (Fig. 16). If this is noted, cannula repositioning can be considered. TEE can also help in identifying the ideal pump speed for a patient on TPD support. When utilizing a “ramp protocol”, where the pump speed is progressively increased intraoperatively, midline interventricular septal position can indicate an appropriate amount of RV support [32].

## 10. Extracorporeal Membrane Oxygenation (ECMO)

The use of ECMO to support patients with refractory shock has increased over the past decade [33]. Forms of ECMO include venovenous (VV ECMO), used primarily to treat respiratory failure, and venoarterial (VA ECMO), used to support patients in cardiogenic shock. VA ECMO is the only form of temporary mechanical support that provides biventricular support and also aids in oxygenation of blood. Different cannulation strategies for ECMO are also available. Central cannulation can be used in post-cardiotomy patients with a venous cannula in the RA and arterial cannula in the aortic arch. Percutaneous peripheral cannulation can also be used, with a variety of configurations. This includes lower extremity vessels such as femoral vein and femoral artery. Upper extremity peripheral cannulations with vessels such as the internal jugual vein and axillary artery are also sometimes utilized to permit increased mobility of the patient. Similar to other forms of mechanical circulatory support, echocardiography can be useful in assessing candidacy for ECMO, monitoring while on support, and weaning of ECMO.

Prior to ECMO cannulation an echocardiographic assessment of the patient should be performed. This can help identify etiologies of hemodynamic collapse which might be reversible without utilizing ECMO. This includes cardiac tamponade or acute valvular pathology. The presence of aortic dissection is a relative contraindication to ECMO cannulation as the arterial cannula can cause further propagation of the dissection flap. Despite this, it can be used as salvage therapy in patients with dissection and no other options, although its use in this setting is associated with high mortality [34, 35]. Pre-existing AR and MR should be identified as both can worsen with the increased afterload seen from VA ECMO.

Cannulation can be performed with guidance of fluorscopy, TTE, or TEE. When TEE is available, it provides the superior spatial resolution and helps identify exact positioning of the guidewires and cannulas during cannulation. The venous cannula is typically positioned in the right atrium. The midesophageal bicaval view is the best view to visualize the right atrium and surrounding structures including SVC, IVC, and interatrial septum. Complications such as migration of the venous cannular across the interatrial septum into the left atrium can be easily identified on TEE [36]. When placed via the femoral artery, the arterial cannula is typically positioned within the descending aorta. When placed via the axillary artery, the arterial cannula is positioned in the aortic arch. Both locations can be visualized with TEE and can help with confirming correct placement during cannulation. Presence of any significant atheromoatous plaque should be noted and relayed to the operator so as to prevent any embolization during the procedure.

Echo is perhaps the most useful tool in monitoring cardiac function when supported by VA ECMO. Other traditional measures of cardiac output such as thermodilution and fick are unreliable and affected by the hemodynamic effects of the ECMO circuit. The arterial outflow cannula will increase the afterload to the LV and therefore it is of paramount importance to ensure that the aortic valve is opening during systole. Failure to do so incrases the risk of LV and aortic root thrombus. To help unload the LV, “venting strategies” are frequently utilized. These include placement of additional devices such as an IABP, Impella, or performing an atrial septostomy, as well as direct LV venting with an additional cannula placed at the apex. As mentioned previously, echo can be helpful in placement and monitoring of these devices as well.

Some authors have described the use of contrast echocardiography to augment images acquired while on ECMO support [37]. The use of contrast can help better assess LV function in patients with poor acoustic windows and also help in identifying any intracardiac thrombi. While contrast microbubbles have been used on rare occasions, there are certain safety issues specific to ECMO that should be noted. There can be accelerated destruction of the microbubbles by the ECMO circuit. More concerning is that the ECMO circuits are designed to detect air bubbles in the system. Contrast microbubbles can activate this alarm which can trigger imminent pump shutdown. Therefore authors have suggested formulating contrast echocardiography protocols specific to ECMO patients, and have a perfusionist or ECMO specialist at bedside when utilizing contrast agents [37]. High mechanical index of the ultrasound beam can be utilized to destroy any remaining bubbles after completing image acquisition.

Finally, echocardiography can be used to help in ECMO weaning. Weaning is considered when there are signs of myocardial recovery. Flows on the circuit can be reduced progressively to 1 to 1.5 L/min while simultaneously using echo to monitor the cardiac response. Echo parameters that have been predictive of successful weaning include LVEF >20–25%, aortic velocity time integral (VTI) >10 cm, and lateral mitral annular systolic wave velocity (S’) >6 cm/sec [38]. Increases in lateral e’ and tricuspid annular S’ velocities have also been demonstrated to predict successful weaning from ECMO [39].

## 11. Echocardiography in the Management of Patients with a Left Ventricular Assist Devices

The rising incidence of advanced heart failure, together with the significant advancements in mechanical circulatory support (MCS), left ventricular assist devices (LVADs) has become a valuable therapeutic option in patients with end-stage heart failure (HF). At present, LVADs are employed as a bridge to transplantation (BTT), as destination therapy (DT), or as a bridge to recovery in whom myocardial recovery is expected [40, 41]. In December 2020, CMS updated their guidelines for LVAD candicacy to include specific clinical parameters and eliminating the intention to treat (BTT, DT) recommedantions [42].

Over the past two to three decades, a large amount of progress has been made in the field of mechanical circulatory support. There has been an increase in the annual number of LVADs implants worldwide with approximately 5000 implanted worldwide per year [43]. In recent years, HeartMate 2, HeartMate 3 and HeartWare are the most commonly utilized continuous-flow LVADs, with HeartMate 3 being implanted exclusively at present [44].

Echocardiography is the most important imaging tool in the clinical assessment and management of LVAD patients, at distinct junctures of their care. Echocardiography is used in preoperative patient selection, intraoperative imaging, and postoperative surveillance, including optimization of LVAD function, evaluation of native myocardial recovery, and troubleshooting of issues pertaining to the LVAD device itself [26].

### 11.1 Candidate Selection

Transthoracic echocardiography (TTE) is frequently the first-line imaging tool employed to screen potential candidates with end-stage HF for LVAD. The goal of the TTE when determining a candidate’s suitability for LVAD is to exclude potential structural and or functional abnormalities that would preclude the patient from surgery [26] (Table 1).

**Table 1.** **Pre-implantation high risk echocardiographic findings [26]**.

Left ventricle	Small LV Size
	Intracardiac thrombus
	Ventricular septal defect.
	LV apical aneurysm
Right ventricle	RV dysfunction
	RV dilatation
Valvular lesions	>Mild AR
	>Moderate MS
	>Moderate TR
	>Moderate PR
Other high-risk findings	PFO
	Aortic pathology
	Mobile intracardiac mass

AR, Aortic regurgitation; LV, Left ventricle; MS, Mitral stenosis; PFO, Patent foramen ovale; PR, Pulmonic regurgitation; RV, Right ventricle; TR, Tricuspid regurgitation.

### 11.2 Assessment of the Left Ventricle (LV)

Accurate measurements of ejection fraction by echocardiography are of paramount importance. Additionally, measurement of the LV internal dimension (LVIDd) at end-diastole from a 2D parasternal long axis image is a critical measurement in the determination of LVAD candidacy [26, 45]. The preoperative measurement can be subsequently compared with the post implantation study to assess the degree of LV unloading. Given the clinical importance of these measurements, performing a 3D assessment of LV volumes and the use of ultrasound enhancing agents can improve the accuracy [26].

In contrast to the majority of patients who are considered for LVAD, some patients with advanced heart failure have small LV cavities (defined by a LVIDd of less than 63 mm), which is associated with an increased 30-day morbidity and mortality rate after LVAD implantation [46]. Small LV cavities can be seen in patients with smaller body habitus or individuals with cardiac amyloidosis.

**Fig. 17.**
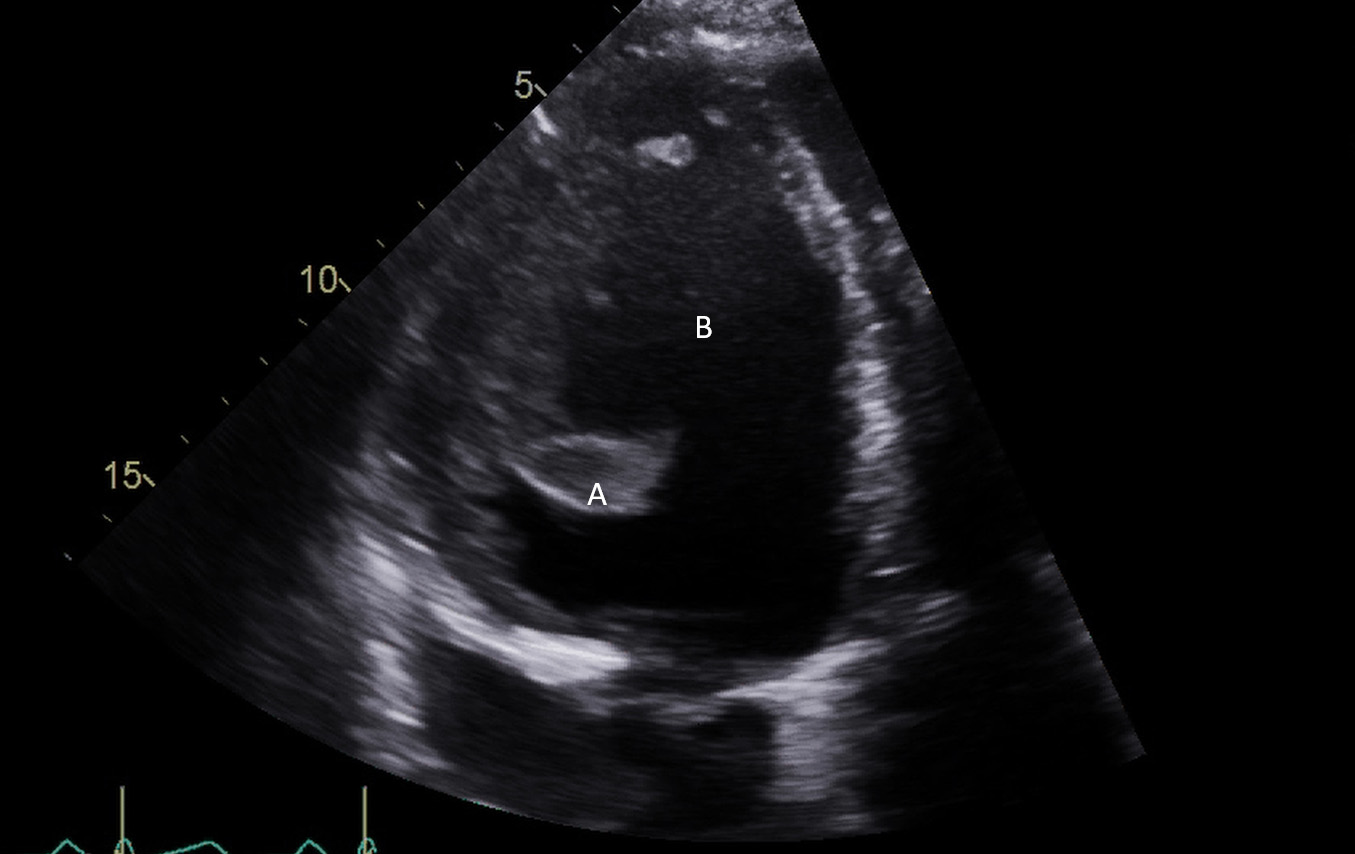
**Apical 4-chamber TTE images demonstrated a large LV thrombus (A) attached to the lateral wall**. (B) LV.

Additionally, an assessment for intracardiac thrombi is of critical importance in the preoperative setting (Fig. 17). While the presence of intracardiac thrombus is not an absolute contraindication, it may increase the risk of embolic events during cannulation [47]. Patients with severely decreased ejection fraction or with a left ventricular aneurysm are at increased risk of developing thrombi. The use of ultrasound enhancing agents can be useful for improved detection of intracardiac thrombi [26]. Transesophageal echocardiography (TEE) may be needed for further delineation of the left atrial appendage in patients with atrial fibrillation.

### 11.3 Assessment of the Right Ventricle (RV)

RV failure requiring the need of an RV assist device is one of the most critical risk factors for morbidity and mortality in patients undergoing LVAD implantation [48]. Post-operative RV dysfunciton remains a significant clinical problem and it’s prediction post LVAD implantation is challenging.

RV dysfunction in the postoperative setting commonly manifests with a decline in end-organ function from low-flow syndrome and increasing central venous pressures. Using a diverse set of defitions in literature RV failure has been described in 3.9–53% of patients receiving LVADs [40, 49].

The worsened prognosis portended by the presence of RV failure after LVAD implantation highlights the importance of accurately identifying patients at risk [40]. If significant right ventricular dysfunction is identified on a preoperative echocardiogram, this may prompt the multidisciplinary heart team to consider potential biventricular mechanical circulatory support (MCS) at the time of surgery [50].

The signs of right ventricular failure on echocardiography include right ventricular systolic dysfunction, RV dilatation, and increased central venous pressures. The latter can be assessed by measuring the size of the inferior vena cava, and its collapsibility. Additionally, moderate or greater tricuspid regurgitation can be seen in patients with significant right ventricular dysfunction [26, 51].

The ASE guidelines recommend 3D echocardiographic assessment of RV volumes in the assessment of RV function. This can be technically challenging in patients with severe cardiomyopathy. Additional surrogates of right ventricular systolic function include RV fractional area change (FAC), tricuspid annular-plane systolic excursion (TAPSE), and RV free wall peak longitudinal strain [26, 27].

With respect to specific clinical parameters predictive of postoperative RV dysfunction in patients undergoing LVAD surgery, few have been described in the literature. Grant *et al*. [52] have illustrated an RV absolute peak longitudinal strain of less than 9.6% as a predictor of RV failure after LVAD implantation. Additionally, Vivo *et al*. [53] have described an increased right-to-left ventricle diameter ratio, of ≥0.75 as a strong predictor of RV failure after LVAD implantation.

### 11.4 Assessment of Pre-Existing Valvular Disease 

In terms of valvular regurgitation, significant aortic regurgitation should be excluded prior to LVAD implantation.

The severity of AR should be quanitifed prior to implantation so that a surgical strategy can be made to address prior to the procedure. When present in patients undergoing LVAD implantation, significant aortic regurgitation creates a circuit of flow in which blood enters the LVAD from the LV and is pumped into the ascending aorta which in turn returns to the LV through the regurgitant aortic valve. This can potentially lead to increased pump flow, reduced stroke volume and high LV pressure [47]. Doppler derived LVOT stroke volume and regurgitant fraction should be calculated routinely when possible. Furthermore, if there is a high clinical suspicion for significant aortic regurgitation, a transesophageal echocardiogram should be considered.

In contrast, significant mitral regurgitation (MR) that is found preoperatively, will often improve after LVAD implantation due to reduction in LV size and filling pressures. This, in turn, improves coaptation of the MV leaflets. As such, any degree of mitral regurgitation is typically acceptable in the LVAD candidate.

With respect to the tricuspid valve, moderate or greater tricuspid regurgitation may indicate significant RV dysfunction and this should be communicated to the multidisciplinary team prior to LVAD implantation. Tricuspid valve repair may be considered the time of surgery [51].

Acute endocarditis is an absolute contraindication to durable LVAD implantation. As such, any independently mobile mass seen during preoperative echocardiographic assessment should be communicated to the team. Any evidence of aneurysmal dilatation of the aorta or even dissection should be further evaluated with multimodality imaging.

**Fig. 18.**
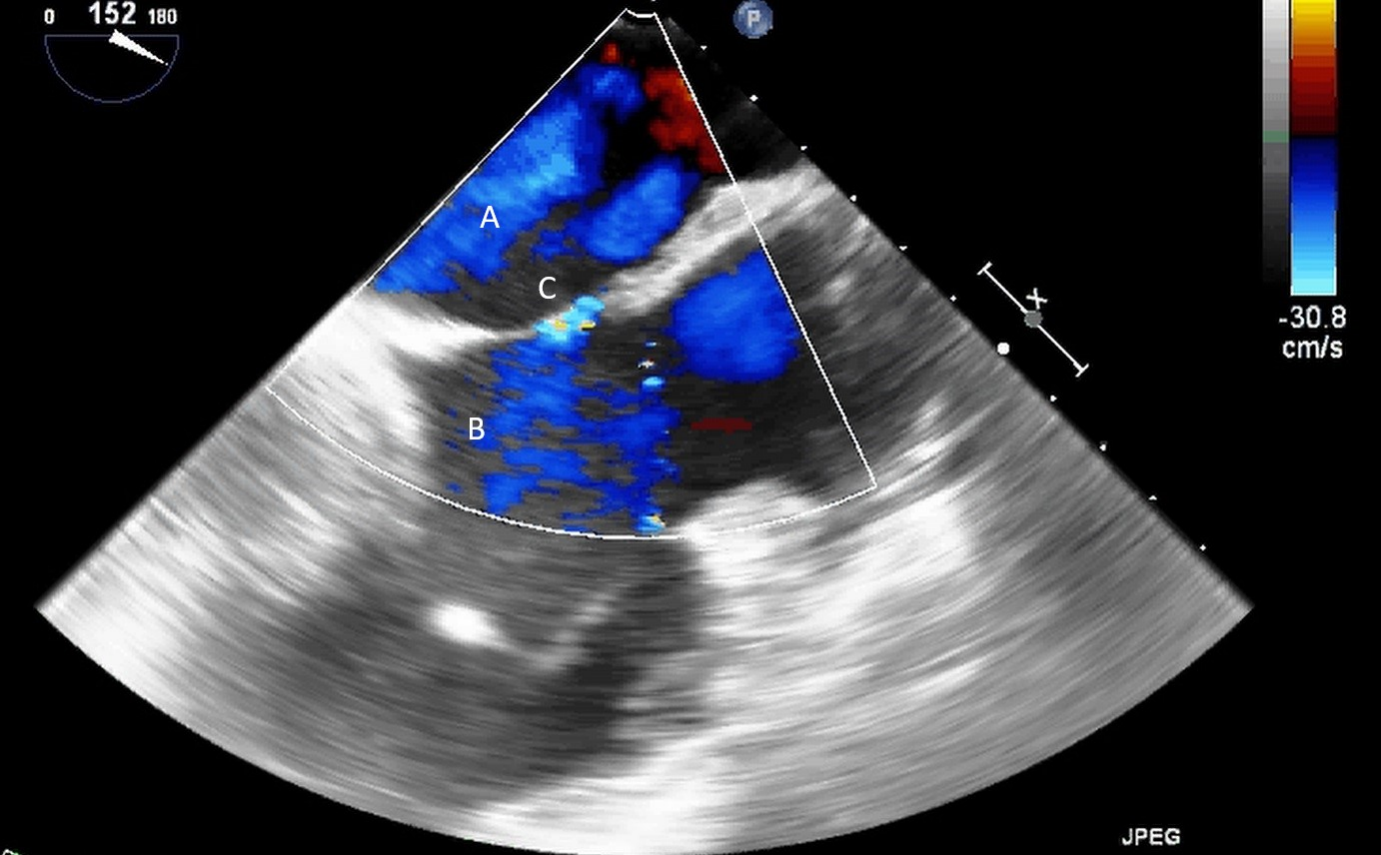
**Transesophageal bi-caval view (Pre-LVAD implantation) demonstrating a left-to-right interatrial shunt by color-flow imaging (C)**. (A) LA. (B) RA.

The prevalence of a patent foramen ovale (PFO) in the U.S. population is approximately 25% [54]. Identification of an interatrial shunt is critically important before LVAD implantation (Fig. 18). Due to the risk of hypoxemia and paradoxical embolization in patients with LVAD, any interatrial communication is typically closed at the time of device implantation [55]. Given the potential risks associated with a PFO in the post LVAD patient, agitated saline and color flow imaging by TEE can be helpful in its identification.

### 11.5 Intraoperative TEE

In the operating room, prior to LVAD placement a thorough TEE should be completed. Significant aortic regurgitation, presence of a patent foramen ovale (PFO) and RV dysfunction should be communicated to the surgical team.

Detection of air bubbles in the immediate post LVAD implantation can be seen on TEE. Inspection for air is critically important as both systemic and coronary embolization can occur down the right coronary artery which can result in right ventricular ischemia, poor hemodynamic affects and on LVAD function.

LVAD activation should lead to LV unloading. A slight leftward interventricular septal (IVS) position indicates adequate LV decompression. Lack of LV decompression in the post implant, rightward shift of the IVS septum should alert the team to the possibility of suboptimal LVAD support, abnormalities with device function or obstruction in the inflow or outflow cannula. In contrast, an extreme leftward shift raises the possibility of excessive unloading due to high pump speed, significant tricuspid regurgitation or right ventricular failure. Intraoperative TEE assessment of the RV function is necessary to determine the need for RVAD support.

**Fig. 19.**
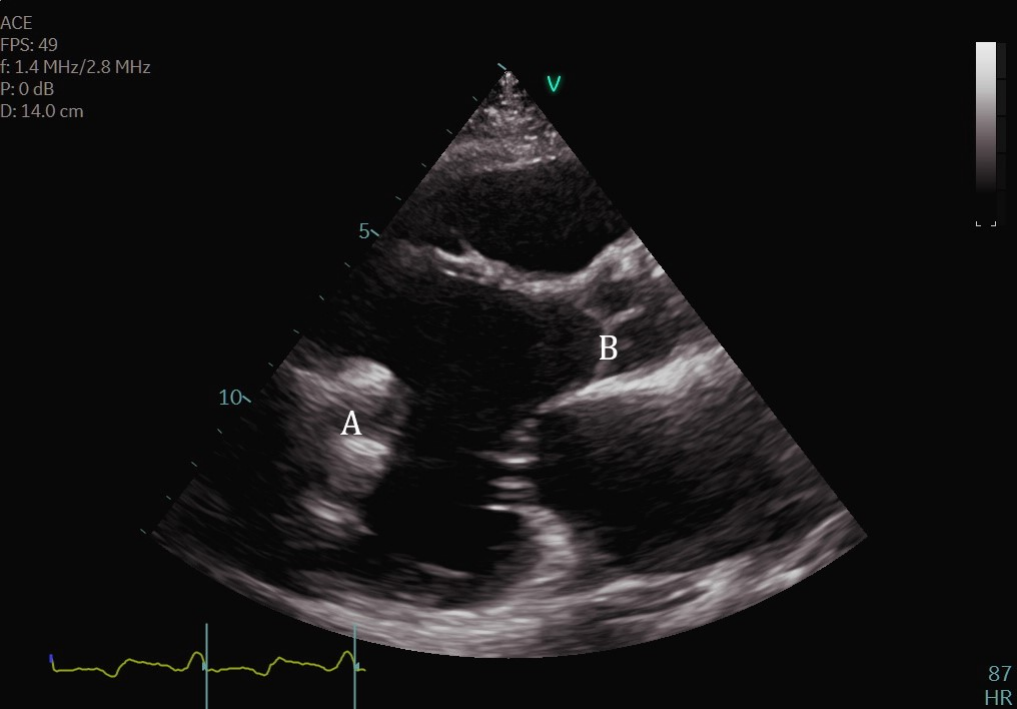
**Transthoracic echocardiogram PLAX image demonstrating inflow cannula at LV apex (A) and AV systolic closure (B)**.

**Fig. 20.**
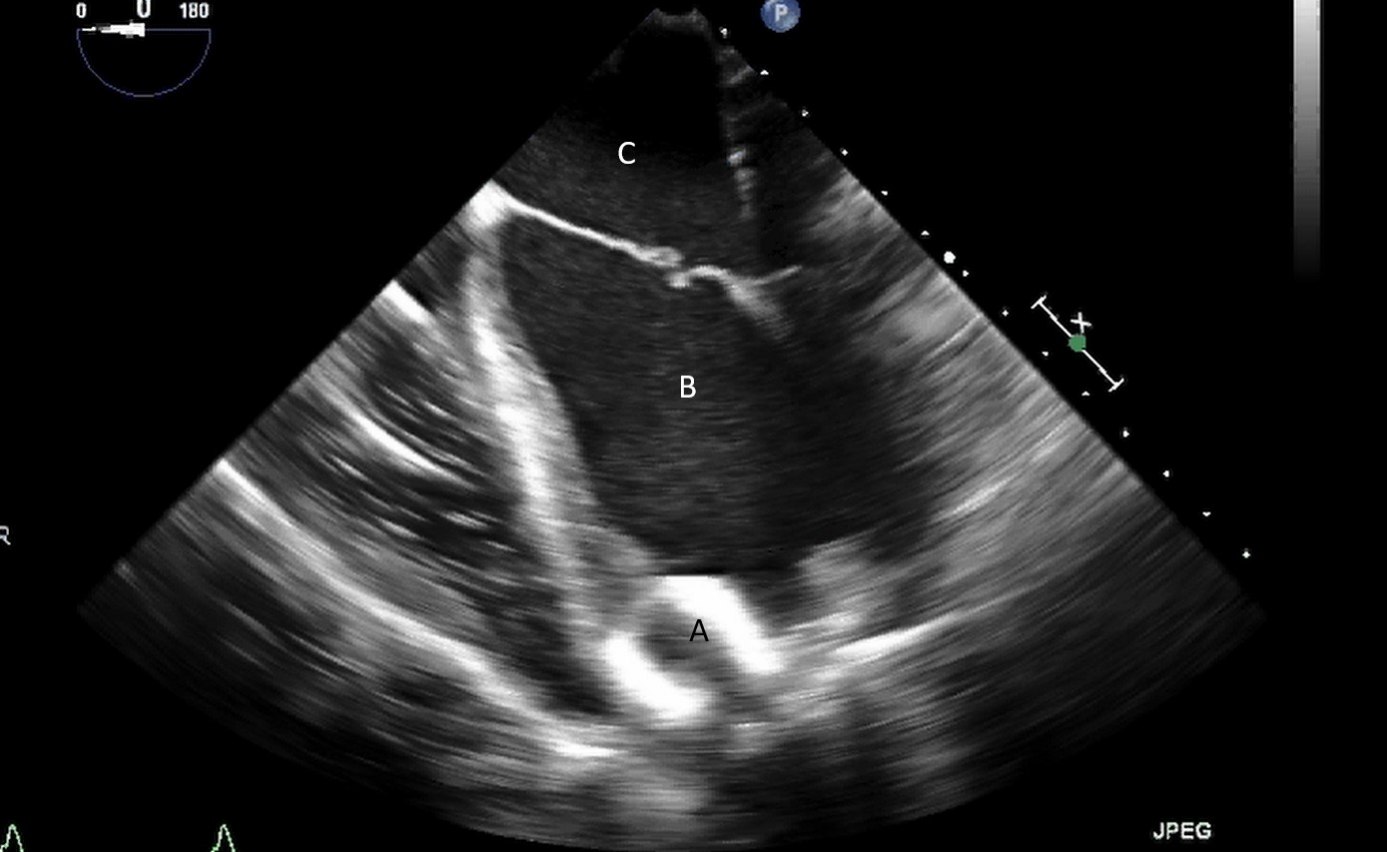
**Transesophageal echocardiogram four chamber image with the LVAD inflow cannula pointing towards the septum (A)**. (B) LV (C) LA.

When examining the inflow cannula of the LVAD, the inflow cannula should be orientated and aligned with the mitral valve [47] (Figs. 19,20). Laminar flow from the ventricle to the device suggests a correctly aligned inflow cannula [56]. Obstruction of the inflow cannula manifests with increased turbulence and elevated doppler velocities [47].

**Fig. 21.**
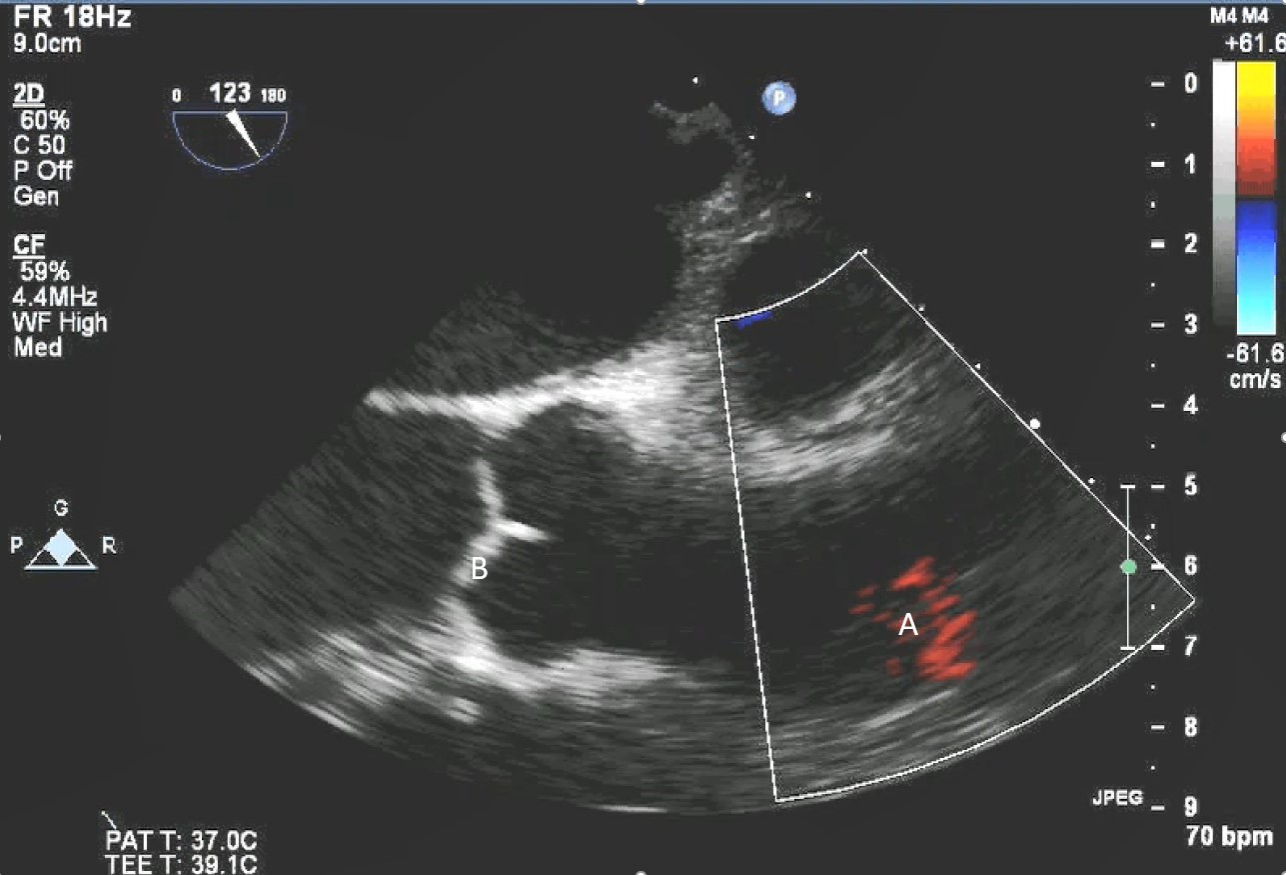
**High-esophageal transesophageal image with the LVAD outflow cannula in the (A) ascending aorta**. (B) Aortic valve.

The outflow cannula is best seen in long axis view of the ascending aorta at the level of the right pulmonary artery [26] (Fig. 21). Velocities greater than 2 m/s from the outflow cannula may suggest obstruction [26]. Multi-modality imaging with computed tomography (CT) can be a useful tool to in the assessment of patency of the outflow cannula.

### 11.6 Postimplantation Evaluation and Troubleshooting

Postimplantation, there are number of key items to evaluate and report on when an LVAD patient receives a TTE (Table 2) [47]. The left ventricular size and function should be reported, as well as the position of the intraventricular septum – midline or shifted either leftward or rightward. Identification and the location of the cannulas should also be noted.

**Table 2.** **Post-operative LVAD surveillance [47]**.

Assessment of biventricular size and function
Assessment of AV morphology, degree of AR and AV opening
Assessment of inflow/outflow cannula location and confirmation of normal velocities (<2 m/s) by doppler
Assessment of Interventriuclar and interatrial septal location
Assessment of the degree of MR and TR
Interrogation of the flow and power of LVAD

The aortic valve should be interrogated and any evidence of aortic insufficiency should be reported [57].

**Fig. 22.**
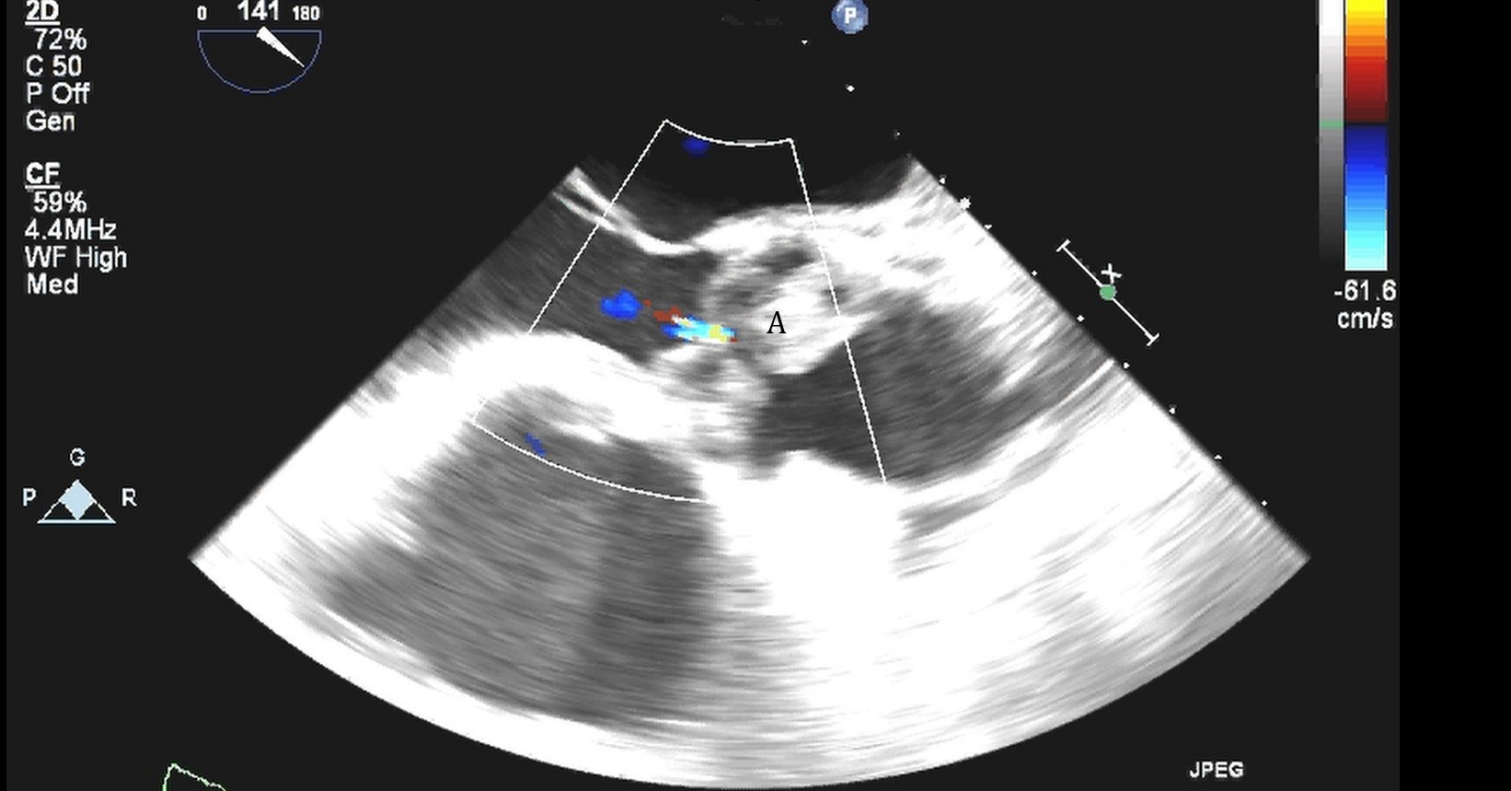
**Transesophageal long axis image demonstrating a large aortic root thrombus (A) with aortic regurgitation**.

In addition, the RV size and function. It is important to comment on if there is evidence of thrombus. With regards to aortic valve opening, it is preferable for the AV to open periodically to prevent permanent closure and thrombosis [47] (Fig. 22). In the event of LVAD malfunction, an AV that opens periodically can assist with LV ejection [44].

Postoperative hemodynamic instability in an LVAD patient carries a specific differential diagnosis. Possible etiologies include hypovolemia, acute RV dysfunction, cardiac tamponade, pulmonary embolism or LVAD malfunction. Echocardiography allows for immediate assessment and detection of the underlying cause of the hemodynamic instability.

Cardiac tamponade and post-LVAD hemorrhage is reported in up to 20% of patients with LVAD, often requiring a return to the operating room for re-exploration [58]. Typical echocardiographic features of tamponade may be masked by the LVAD but potential echocardiographic clues to the diagnosis include compression of the right and left atria associated with a reduction in biventricular size [26].

Small RV and LV cavities in the absence of additional findings suggests hypovolemia. TTE findings of a poorly functioning, dilated RV with associated functional TR should raise the suspicion for acute RV dysfunction. The LV may also be collapsed with inflow cannula obstruction [47].

## 12. The Role of Echocardiography in Patients with Total Artificial Heart (TAH)

**Fig. 23.**
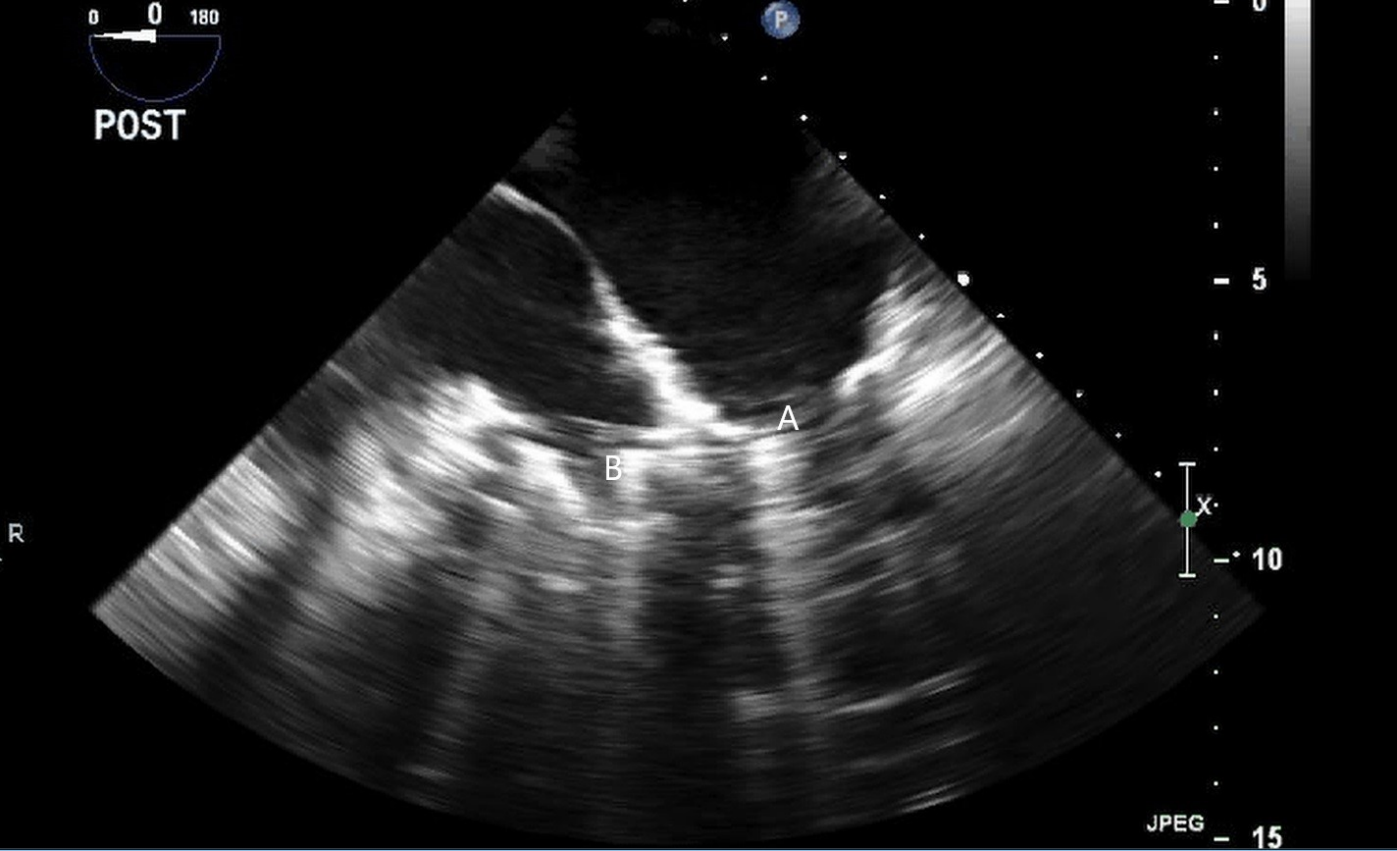
**Mid-esophageal 4-chamber view with both Medtronic-Hall valves; Mitral (A) and Tricuspid (B) of the TAH visualized**.

**Fig. 24.**
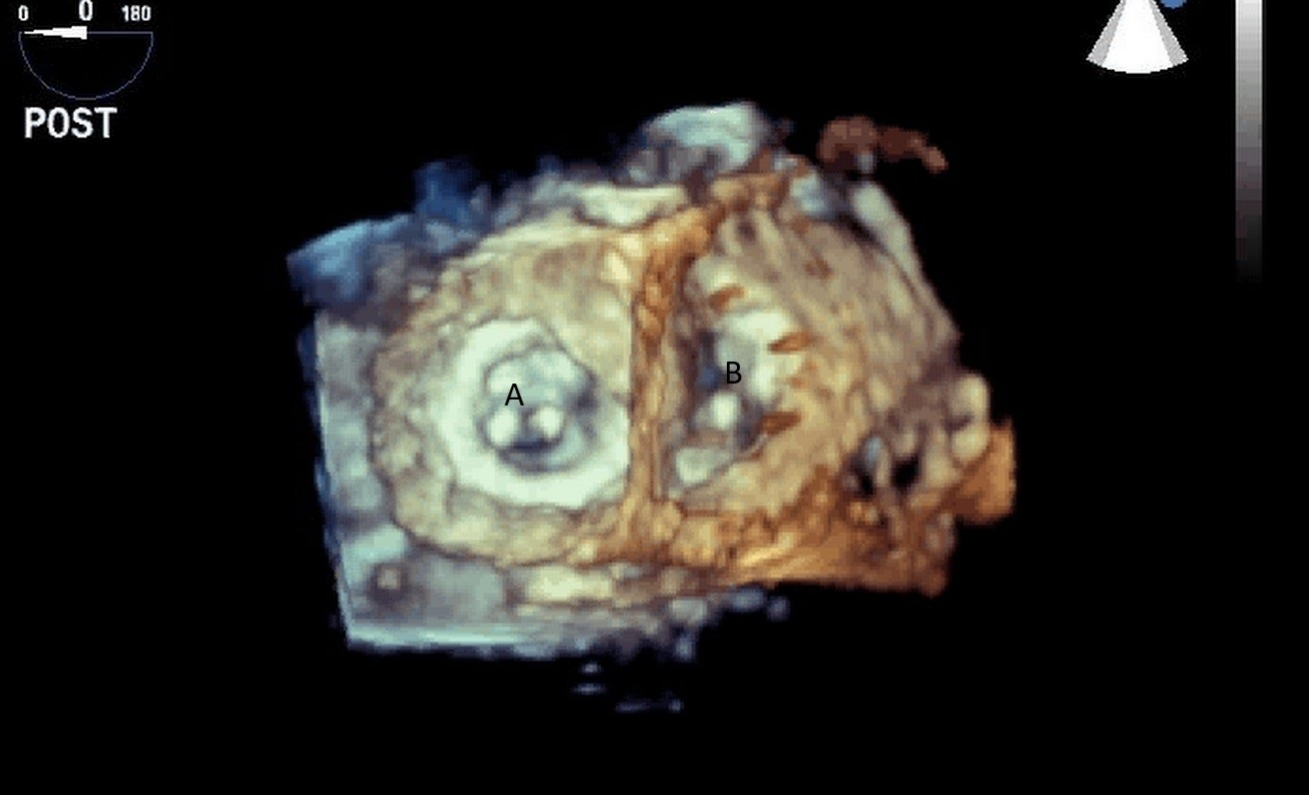
**Three-dimensional transesophageal echocardiographic image of the Medtronic-Hall valves in the mitral (A) and tricuspid valve (B) positions**.

For patients who are not candidates for LVAD due to RV dysfunction, total artificial heart (TAH) is an alternative option for mechanical circulatory support. The SynCardia device has been FDA approved for advanced HF since 2004. It is a biventricular pneumatic pulsatile device comprising of two artificial ventricles. Each ventricles has an inflow (Figs. 23,24) and outflow valve (Medtronic-Hall, single tilting disc valve) [59].

Similarly to the intraoperative assessment in the LVAD patient, a comprehensive study should be performed prior to TAH to assess for pulmonary venous abnormalities, intracardiac shunt, intracardiac thrombus and to identify the location of the central venous catheter within in the right atrium to ensure it does not interfere with the TAH inflow valve [60]. Additionally, assessment of the IVC is necessary to establish a baseline size prior to future studies for evaluation of caval compression.

Post implantation, confirmation of functioning discs, evaluation for caval compression and patency of pulmonary veins are key items to investigate [59].

## 13. Summary

Echocardiography is a valuable tool in the identification of suitable candidates for MCS. Both transesophageal and transthoracic modalities may assist with the placement, optimization, troubleshooting and weaning of the support device implimented. With updated guidelines for cardiogenic shock and advanced HF, the number of patients eligible for MCS is likely to continue to rise and echocardiography will play a critical role in the evaluation and management of this population. As new support devices come to market, the echocardiography knowledge base will need to evolve to include parameters for evaluation, monitoring and optimization of these new devices.

## References

[1] Luis SA, Chan J, Pellikka PA (2019). Echocardiographic Assessment of Left Ventricular Systolic Function: An Overview of Contemporary Techniques, Including Speckle-Tracking Echocardiography. *Mayo Clinic Proceedings*.

[2] Krishna M, Zacharowski K (2009). Principles of intra-aortic balloon pump counterpulsation. *Continuing Education in Anaesthesia Critical Care & Pain*.

[3] Butala B, Yu R, Schorr R, Gologorsky E (2020). Periprocedural Dynamics of Aortic Regurgitation in Patients Supported with an Impella Left Ventricular Assist Device. *Journal of Cardiothoracic and Vascular Anesthesia*.

[4] Weaver H, Farid S, Nashef S, Catarino P (2017). Use of Intraaortic Balloon Pumps in Acute Type a Aortic Dissection. *The Annals of Thoracic Surgery*.

[5] Hiraoka A, Saku K, Nishikawa T, Sunagawa K (2021). A case report of unexpected right-to-left shunt under mechanical support for post-infarction ventricular septal defect: evaluation with haemodynamic simulator. *European Heart Journal - Case Reports*.

[6] MacKay EJ, Patel PA, Gutsche JT, Weiss SJ, Augoustides JG (2017). Contemporary Clinical Niche for Intra-Aortic Balloon Counterpulsation in Perioperative Cardiovascular Practice: an Evidence-Based Review for the Cardiovascular Anesthesiologist. *Journal of Cardiothoracic and Vascular Anesthesia*.

[7] Rehfeldt KH, Click RL (2003). Intraoperative transesophageal echocardiographic imaging of an intra-aortic balloon pump placed via the ascending aorta. *Journal of Cardiothoracic and Vascular Anesthesia*.

[8] Rosenbaum AN, Jain CC, Shadrin IY, El Hajj SC, El Sabbagh A, Behfar A (2021). Percutaneous Axillary Intra-aortic Balloon Pump Insertion Technique as Bridge to Advanced Heart Failure Therapy. *ASAIO Journal*.

[9] Klopman MA, Chen EP, Sniecinski RM (2011). Positioning an Intraaortic Balloon Pump Using Intraoperative Transesophageal Echocardiogram Guidance. *Anesthesia & Analgesia*.

[10] Papaioannou TG, Stefanadis C (2005). Basic Principles of the Intraaortic Balloon Pump and Mechanisms Affecting its Performance. *ASAIO Journal*.

[11] Takamura T, Dohi K, Satomi A, Mori K, Moriwaki K, Sugimoto T (2012). Intra-aortic balloon pump induced dynamic left ventricular outflow tract obstruction and cardiogenic shock after very late stent thrombosis in the left anterior descending coronary artery. *Journal of Cardiology Cases*.

[12] Glazier JJ, Kaki A (2019). The Impella Device: Historical Background, Clinical Applications and Future Directions. *International Journal of Angiology*.

[13] Crowley J, Cronin B, Essandoh M, D’Alessandro D, Shelton K, Dalia AA (2019). Transesophageal Echocardiography for Impella Placement and Management. *Journal of Cardiothoracic and Vascular Anesthesia*.

[14] Pieri M, Pappalardo F (2020). Bedside insertion of impella percutaneous ventricular assist device in patients with cardiogenic shock. *International Journal of Cardiology*.

[15] Patel KM, Sherwani SS, Baudo AM, Salvacion A, Herborn J, Soong W (2012). The Use of Transesophageal Echocardiography for Confirmation of Appropriate Impella 5.0 Device Placement. *Anesthesia & Analgesia*.

[16] Elhussein TA, Hutchison SJ (2014). Acute Mitral Regurgitation: Unforeseen New Complication of the Impella LP 5.0 Ventricular Assist Device and Review of Literature. *Heart, Lung and Circulation*.

[17] Mizuno A, Kawamoto S, Uda S, Tatsumi K, Takeda C, Tanaka T (2021). A case of left ventricular free wall rupture after insertion of an IMPELLA® left ventricular assist device diagnosed by transesophageal echocardiography. *JA Clinical Reports*.

[18] Cardozo S, Ahmed T, Belgrave K (2015). Impella Induced Massive Hemolysis: Reemphasizing Echocardiographic Guidance for Correct Placement. *Case Reports in Cardiology*.

[19] Tschöpe C, Spillmann F, Potapov E, Faragli A, Rapis K, Nelki V (2021). The ”TIDE”-Algorithm for the Weaning of Patients With Cardiogenic Shock and Temporarily Mechanical Left Ventricular Support With Impella Devices. A Cardiovascular Physiology-Based Approach. *Frontiers in Cardiovascular Medicine*.

[20] Kar B, Adkins LE, Civitello AB, Loyalka P, Palanichamy N, Gemmato CJ (2006). Clinical experience with the TandemHeart percutaneous ventricular assist device. *Texas Heart Institute Journal*.

[21] Gilotra NA, Stevens GR (2014). Temporary Mechanical Circulatory Support: a Review of the Options, Indications, and Outcomes. *Clinical Medicine Insights: Cardiology*.

[22] Kooshkabadi M, Kalogeropoulos A, Babaliaros VC, Lerakis S (2008). Transesophageal guided left atrial positioning of a percutaneous ventricular assist device. *European Journal of Echocardiography*.

[23] Pretorius M, Hughes AK, Stahlman MB, Saavedra PJ, Deegan RJ, Greelish JP (2006). Placement of the TandemHeart® Percutaneous Left Ventricular Assist Device. *Anesthesia & Analgesia*.

[24] Ballal RS, Mahan EF, Nanda NC, Dean LS (1990). Utility of transesophageal echocardiography in interatrial septal puncture during percutaneous mitral balloon commissurotomy. *The American Journal of Cardiology*.

[25] De Ponti R, Cappato R, Curnis A, Della Bella P, Padeletti L, Raviele A (2006). Trans-Septal Catheterization in the Electrophysiology Laboratory. *Journal of the American College of Cardiology*.

[26] Stainback RF, Estep JD, Agler DA, Birks EJ, Bremer M, Hung J (2015). Echocardiography in the Management of Patients with Left Ventricular Assist Devices: Recommendations from the American Society of Echocardiography. *Journal of the American Society of Echocardiography*.

[27] Rudski LG, Lai WW, Afilalo J, Hua L, Handschumacher MD, Chandrasekaran K (2010). Guidelines for the Echocardiographic Assessment of the Right Heart in Adults: a Report from the American Society of Echocardiography. *Journal of the American Society of Echocardiography*.

[28] Morris DA, Krisper M, Nakatani S, Köhncke C, Otsuji Y, Belyavskiy E (2017). Normal range and usefulness of right ventricular systolic strain to detect subtle right ventricular systolic abnormalities in patients with heart failure: a multicentre study. *European Heart Journal – Cardiovascular Imaging*.

[29] Pieri M, Pappalardo F (2018). Impella RP in the Treatment of Right Ventricular Failure: what we Know and where we Go. *Journal of Cardiothoracic and Vascular Anesthesia*.

[30] Jain A, Al-Ani M, Arnaoutakis G, Alviar C, Vilaro J (2019). Troubleshooting right ventricular failure: role of transesophageal echocardiography in assessing impella rp ® position. *Journal of the American College of Cardiology*.

[31] Gomez-Abraham JA, Brann S, Aggarwal V, O’Neill B, Alvarez R, Hamad E (2018). Use of Protek Duo Cannula (RVAD) for Percutaneous Support in Various Clinical Settings. a Safe and Effective Option. *The Journal of Heart and Lung Transplantation*.

[32] Ravichandran AK, Baran DA, Stelling K, Cowger JA, Salerno CT (2018). Outcomes with the Tandem Protek Duo Dual-Lumen Percutaneous Right Ventricular Assist Device. *ASAIO Journal*.

[33] Gerke AK, Tang F, Cavanaugh JE, Doerschug KC, Polgreen PM (2015). Increased trend in extracorporeal membrane oxygenation use by adults in the United States since 2007. *BMC Research Notes*.

[34] Hou JY, Wang CS, Lai H, Sun YX, Li X, Zheng JL (2021). Veno-Arterial Extracorporeal Membrane Oxygenation for Patients Undergoing Acute Type A Aortic Dissection Surgery: A Six-Year Experience. *Frontiers in Cardiovascular Medicine*.

[35] Sultan I, Habertheuer A, Wallen T, Siki M, Szeto W, Bavaria JE (2017). The role of extracorporeal membrane oxygenator therapy in the setting of Type a aortic dissection. *Journal of Cardiac Surgery*.

[36] Giraud R, Banfi C, Bendjelid K (2018). Echocardiography should be mandatory in ECMO venous cannula placement. *European Heart Journal - Cardiovascular Imaging*.

[37] Bennett CE, Tweet MS, Michelena HI, Schears GJ, Mulvagh SL (2017). Safety and Feasibility of Contrast Echocardiography for ECMO Evaluation. *JACC: Cardiovascular Imaging*.

[38] Aissaoui N, Luyt C, Leprince P, Trouillet J, Léger P, Pavie A (2011). Predictors of successful extracorporeal membrane oxygenation (ECMO) weaning after assistance for refractory cardiogenic shock. *Intensive Care Medicine*.

[39] Kim D, Jang WJ, Park TK, Cho YH, Choi J, Jeon E (2021). Echocardiographic Predictors of Successful Extracorporeal Membrane Oxygenation Weaning after Refractory Cardiogenic Shock. *Journal of the American Society of Echocardiography*.

[40] Craig ML (2011). Management of Right Ventricular Failure in the Era of Ventricular Assist Device Therapy. *Current Heart Failure Reports*.

[41] Rose EA, Gelijns AC, Moskowitz AJ, Heitjan DF, Stevenson LW, Dembitsky W (2001). Long-Term Use of a Left Ventricular Assist Device for End-Stage Heart Failure. *New England Journal of Medicine*.

[42] CMS (2020). Artificial Hearts and related devices, including Ventricular Assist Devices for Bridge-to-Transplant and Destination Therapy. https://www.cms.gov/medicare-coverage-database/view/ncacal-decision-memo.aspx?proposed=Y&NCAId=298.

[43] Miller L, Birks E, Guglin M, Lamba H, Frazier OH (2019). Use of Ventricular Assist Devices and Heart Transplantation for Advanced Heart Failure. *Circulation Research*.

[44] Bouchez S, Van Belleghem Y, De Somer F, De Pauw M, Stroobandt R, Wouters P (2019). Haemodynamic management of patients with left ventricular assist devices using echocardiography: the essentials. *European Heart Journal - Cardiovascular Imaging*.

[45] Mitchell C, Rahko PS, Blauwet LA, Canaday B, Finstuen JA, Foster MC (2019). Guidelines for Performing a Comprehensive Transthoracic Echocardiographic Examination in Adults: Recommendations from the American Society of Echocardiography. *Journal of the American Society of Echocardiography*.

[46] Topilsky Y, Oh JK, Shah DK, Boilson BA, Schirger JA, Kushwaha SS (2011). Echocardiographic Predictors of Adverse Outcomes after Continuous Left Ventricular Assist Device Implantation. *JACC: Cardiovascular Imaging*.

[47] Ammar KA, Umland MM, Kramer C, Sulemanjee N, Jan MF, Khandheria BK (2012). The ABCs of left ventricular assist device echocardiography: a systematic approach. *European Heart Journal - Cardiovascular Imaging*.

[48] Deng MC, Edwards LB, Hertz MI, Rowe AW, Keck BM, Kormos R (2005). Mechanical circulatory support device database of the International Society for Heart and Lung Transplantation: third annual report–2005. *The Journal of Heart and Lung Transplantation*.

[49] Ali HR, Kiernan MS, Choudhary G, Levine DJ, Sodha NR, Ehsan A (2020). Right Ventricular Failure Post-Implantation of Left Ventricular Assist Device: Prevalence, Pathophysiology, and Predictors. *ASAIO Journal*.

[50] Fitzpatrick JR, Frederick JR, Hiesinger W, Hsu VM, McCormick RC, Kozin ED (2009). Early planned institution of biventricular mechanical circulatory support results in improved outcomes compared with delayed conversion of a left ventricular assist device to a biventricular assist device. *The Journal of Thoracic and Cardiovascular Surgery*.

[51] Feldman D, Pamboukian SV, Teuteberg JJ, Birks E, Lietz K, Moore SA (2013). The 2013 International Society for Heart and Lung Transplantation Guidelines for mechanical circulatory support: Executive summary. *The Journal of Heart and Lung Transplantation*.

[52] Grant ADM, Smedira NG, Starling RC, Marwick TH (2012). Independent and Incremental Role of Quantitative Right Ventricular Evaluation for the Prediction of Right Ventricular Failure after Left Ventricular Assist Device Implantation. *Journal of the American College of Cardiology*.

[53] Vivo RP, Cordero-Reyes AM, Qamar U, Garikipati S, Trevino AR, Aldeiri M (2013). Increased right-to-left ventricle diameter ratio is a strong predictor of right ventricular failure after left ventricular assist device. *The Journal of Heart and Lung Transplantation*.

[54] Waldenberger F, Kim Y, Laycock S, Meyns B, Flameng W (1996). Effects of failure of the right side of the heart and increased pulmonary resistance on mechanical circulatory support with use of the miniaturized HIA-VAD displacement pump system. *The Journal of Thoracic and Cardiovascular Surgery*.

[55] Kilger E, Strom C, Frey L, Felbinger TW, Pichler B, Tichy M (2000). Intermittent atrial level right-to-left shunt with temporary hypoxemia in a patient during support with a left ventricular assist device. *Acta Anaesthesiologica Scandinavica*.

[56] Horton SC, Khodaverdian R, Chatelain P, McIntosh ML, Horne BD, Muhlestein JB (2005). Left Ventricular Assist Device Malfunction. *Journal of the American College of Cardiology*.

[57] George A, Butt T, MacGowan G, Patangi S, Pauli H, O’Leary D (2013). Management issues during HeartWare left ventricular assist device implantation and the role of transesophageal echocardiography. *Annals of Cardiac Anaesthesia*.

[58] Topilsky Y, Price TN, Atchison FW, Joyce LD (2011). Atypical tamponade hemodynamic in a patient with temporary left ventricular assist device. *Interactive CardioVascular and Thoracic Surgery*.

[59] Mizuguchi KA, Padera RF, Kowalczyk A, Doran MN, Couper GS, Fox AA (2013). Transesophageal Echocardiography Imaging of the Total Artificial Heart. *Anesthesia & Analgesia*.

[60] Fine NM, Gopalan RS, Arabia FA, Kushwaha SS, Chandrasekaran K (2014). Intraoperative transesophageal echocardiographic guidance of total artificial heart implantation. *The Journal of Heart and Lung Transplantation*.

